# The phenomenon of red and yellow autumn leaves: Hypotheses, agreements and disagreements

**DOI:** 10.1111/jeb.14069

**Published:** 2022-08-16

**Authors:** Simcha Lev‐Yadun

**Affiliations:** ^1^ Department of Biology & Environment, Faculty of Natural Sciences University of Haifa Tivon Israel

**Keywords:** anthocyanins, aphids, aposematism, carotenoids, co‐evolutionary hypothesis, herbivory, photoprotection

## Abstract

Yellow and red autumn leaves are typical of many temperate/boreal woody plants. Since the 19^th^ century, it has been either considered the non‐functional outcome of chlorophyll degradation that unmasks the pre‐existing yellow and red pigments or that the de novo synthesis of red anthocyanins in autumn leaves indicated that it should have a physiological function, although it was commonly ignored. Defending free amino acids and various other resources released especially following the breakdown of the photosynthetic system, and mobilizing them for storage in other organs before leaf fall, is the cornerstone of both the physiological and anti‐herbivory hypotheses about the functions of yellow and red autumn leaf colouration. The complicated phenomenon of conspicuous autumn leaf colouration has received significant attention since the year 2000, especially because ecologists started paying attention to its anti‐herbivory potential. The obvious imperfection of the hypotheses put forth in several papers stimulated many other scientists. Hot debates among physiologists, among ecologists, and between physiologists and ecologists have been common since the year 2000, first because the various functions of yellow and red autumn leaf colouration are non‐exclusive, and second because many scientists were trained to focus on a single subject. Here, I will review the debates, especially between the photoprotective and the anti‐herbivory hypotheses, and describe both the progress in their understanding and the required progress.

## Graphical abstract

Yellow and red autumn leaves typical to temperate/boreal trees defend important resources released following the photosynthetic system breakdown, and mobilized for storage to be used in the next spring. This coloration, especially red, defends from excess light and oxidative stress and against herbivores. I describe the hypotheses, findings and disagreements about the functions and evolution of yellow/red autumn leaves.

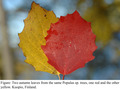

## 1 INTRODUCTION

While reviewing the proposed functions and evolution of autumn leaf colouration in woody plants of the temperate/boreal zones, I begin by addressing several factual and theoretical issues, which bear on the understanding of the potential physiological and anti‐herbivory functions of autumnal leaf colouration. Reviewing only the recent (2019–2022) debate without outlining the background and history of the subject will be partly meaningless if not misleading, especially for the many who are newcomers to this issue. After describing the basics of the theoretical arena in which the plants, their herbivores and pathogens, and their researchers play, I will refer to the three waves of research, discussions and progress in the study of yellow and red autumn leaf colouration since the year 2000: the first wave in the years 2000–2009, the second in the years 2009–2018 and the current wave of papers, which started in the year 2019, with a review that focused on physiology and paid only little attention to anti‐herbivory (Renner & Zohner, 2019).

I stress that when I deal with a multifunctional character such as yellow and red autumn leaf colouration, I fully agree with Endler (1981), who commented concerning the functions of animal colouration: ‘we must be careful not to assume that because we have found one apparent function to a colour pattern, it necessarily means that we have a complete explanation’. Unfortunately, some of the scientists who study autumn leaf colouration stick to a single explanation and ignore clear evidence of the simultaneous operation of other mechanisms.

When I first learned, in a geobotany course in 1978 during my B.Sc. studies, about the dramatic phenomenon of red or yellow autumn leaves in the temperate region, the explanation we obtained was that these conspicuous colours appear when the chlorophylls are degraded in order to mobilize their amino acids and other resources for storage in the branches and trunks before leaf shed, to be used for new growth or flowering in the following spring, and that these colours existed in the leaves all the time but were masked by green chlorophylls. This was a common belief, originating in the marginal theoretical and experimental attention given to the phenomenon of autumn leaf colouration, which caused the common ignorance of even the little physiological information that was known about the functions of red autumn leaf colouration for more than a century. Already in the 19^th^ century, it was known that certain red autumn leaves may sometimes be warmer than leaves of other colours, that the production of red leaf colouration in temperate deciduous trees is commonly associated with low temperatures, with wounding or disease, and with autumnal leaf fall, and that anthocyanins may defend the photosynthetic system from high light levels (Haberlandt, 1914; Lee, 2002, 2012; Lee & Gould, 2002; Wheldale, 1916; and citations therein), but this was not common knowledge for most botanists. This low level of understanding did not change much for decades (see Büsgen, 1929; Kramer & Kozlowski, 1979).

**FIGURE 1:**
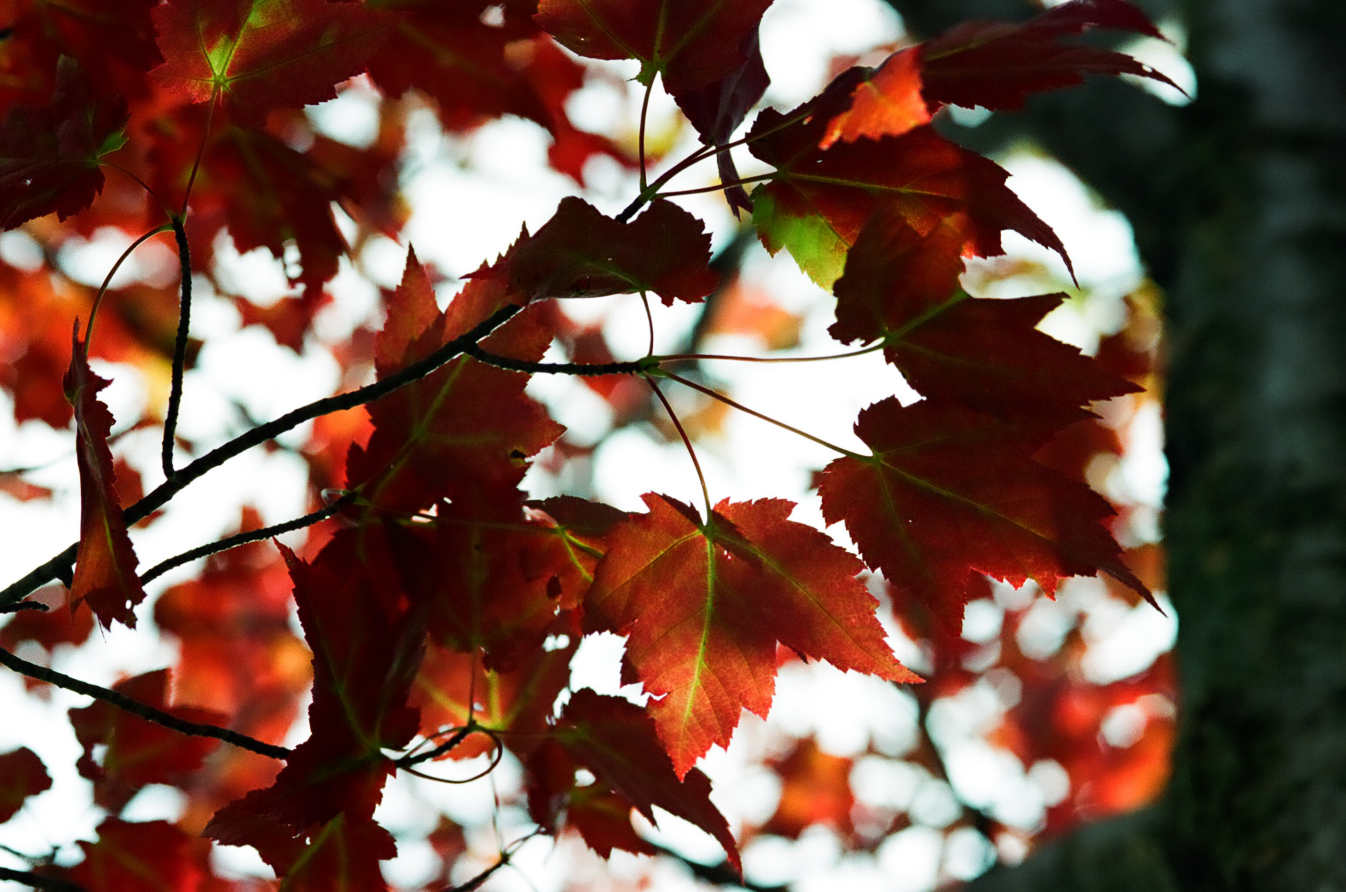
Red autumn leaves, *Acer* sp., Upstate New York

**FIGURE 2:**
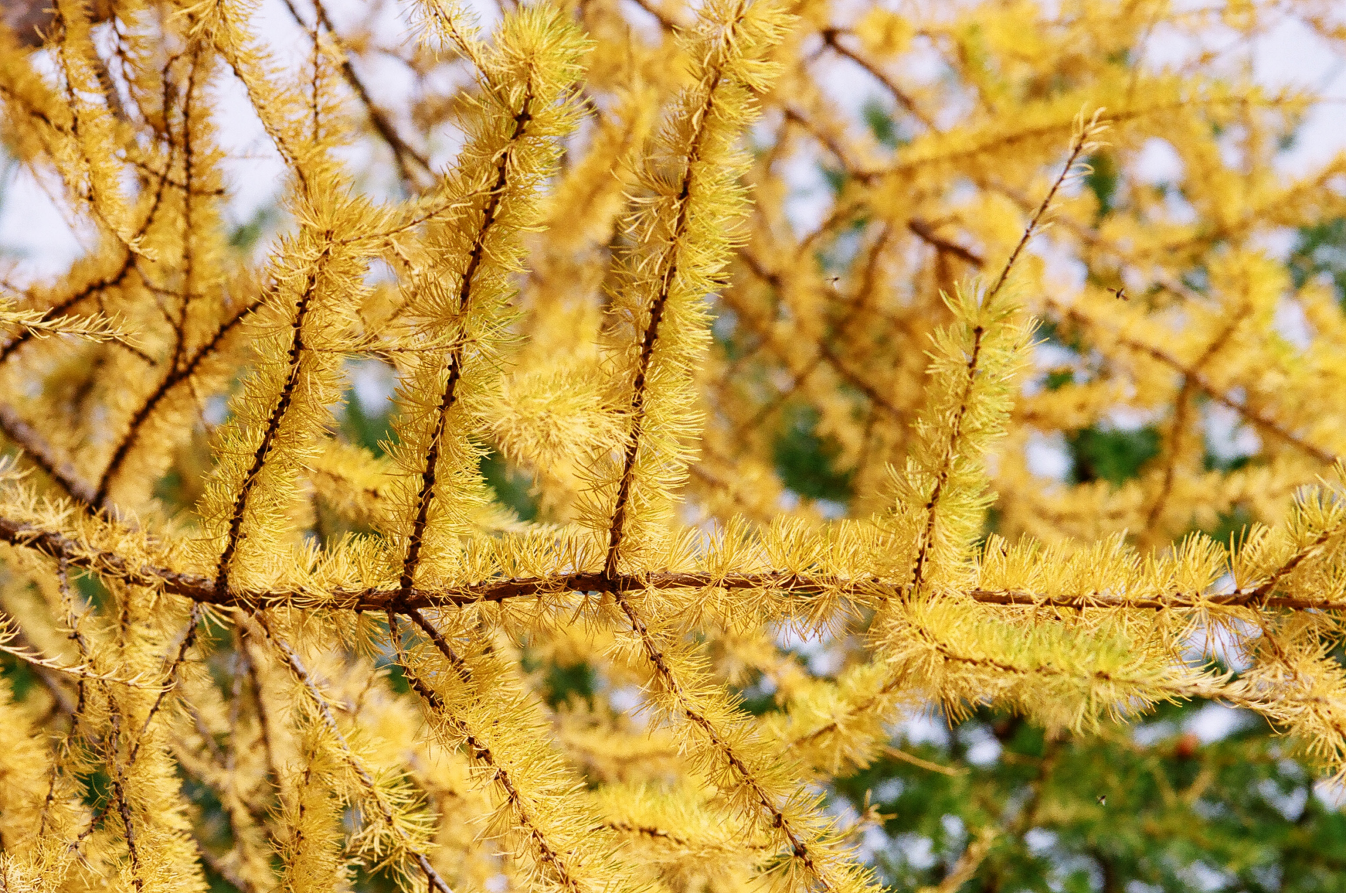
Yellow *Larix sibirica* leaves, Finland

Yellow and red autumn leaves of many woody dicotyledons and a few conifers (Figures 1 and 2) dominate huge areas in the temperate/boreal zones of the world (Archetti, 2009a; Axelrod, 1966; Lee, 2002) and smaller areas in subtropical climates, where trees that have evolved in colder habitats exist because of various historical, topographical and biotic reasons, especially at higher elevations where autumn and winter temperatures are low (e.g. Lev‐Yadun et al., 2012). In eastern North America, in South America and in east Asia, autumn landscapes are visually dominated by many tree, shrub and climber species with red autumn leaves (e.g. Hoch et al., 2001; Lee et al., 2003).

Various physiological adaptations to environmental constraints led to the evolution of the deciduous habit starting about 150 million years ago (e.g. Addicott, 1982; Axelrod, 1966). Thus, the current colourful autumn leaf phenomenon in the temperate/boreal zones has roots in much earlier periods with different climates, floras and faunas, that is especially in the Tertiary but even in earlier geological epochs and under both warmer and cooler climates (see Lev‐Yadun & Holopainen, 2009). Yellow‐senescing leaves in considerable amounts may also occur in tropical zones but not only in autumn, and the timing may be either taxon‐specific or related to the dry winter or to other stressors (Richards, 1996). Although we know that red autumn leaves are much more common in the landscapes of North America, South America, China and Japan than in northern Europe or in Europe in general (Archetti, 2009a; Holopainen & Peltonen, 2002; Lev‐Yadun & Holopainen, 2009), we do not yet have the complete list of deciduous woody species with red autumn leaves in China and Japan. Therefore, analysing the agents that cause the evolution of red autumn leaves, while considering the number of east Asian species in various European or American arboreta, or in various publications, is problematic from the outset. I know from personal communications with Chinese and Japanese scientists that they are currently trying to compile full lists of woody species with red autumn leaves for their countries, but the data do not exist yet. Lev‐Yadun and Holopainen (2009) examined the natural distribution of each of the 290 tree and shrub species with red autumn leaves listed in Archetti et al. (2009a) and found that most of them grow in North America and east Asia. There are at least 89 such species in a subset of the woody flora of North America (e.g. Lee et al., 2003) and at least 152 such species in east Asia (Lev‐Yadun & Holopainen, 2009). However, the criteria used for classifying a species as one with red autumn leaves differed among scientists, and this problem will be explained in more detail in the relevant section of this essay. In any case, I suggest to classify all the species that have red autumn leaves, but not because they are damaged, as red ones, even if they also produce some yellow autumn leaves.

When studying probably more than 1000 temperate/boreal deciduous woody species from several continents that express yellow or red autumn leaf colours, the origin of those species and their evolutionary history, as well as their specific past ecology or ecologies in their native areas, should be considered. The climatic differences between the tropical/subtropical and the temperate/boreal regions as we know them today, and the related biological adaptations have developed gradually following the breakdown of the supercontinent Pangaea and the subsequent closure of the huge ocean Panthalassa, a process that took more than 100 million years. The current land biota, with its sharp differences between the adaptations to warm tropical and subtropical regions, and to the cold temperate and arctic regions, is a relatively new phenomenon in geological and evolutionary timescales, after a very long warm period, with much smaller thermal gradients between the tropical and polar regions than those that we are familiar with today (see Axelrod, 1966; Graham, 1993; Manchester, 1999; Tiffney, 1985). Significant phases of cooling and glaciation alternating with warmer phases began in the mid‐Tertiary, c. 35 million years ago, a process that culminated in the Pleistocene, which started about 2.6 million years ago, with its four major glaciation waves and interglacial warm periods (Imbrie & Palmer‐Imbrie, 1979; Tiffney, 1985; Zachos et al., 2001). Such dramatic climatic changes and their biological consequences were selected for various physiological and ecological adaptations, including adaptations to cold environments with strong seasonality (e.g. Addicott, 1982; Axelrod, 1966; Delcourt & Delcourt, 1993; Graham, 1993; Stebbins, 1974; Takhtajan, 1991; Tiffney, 1985; Wen, 1999).

Four times during the Pleistocene, huge areas in Asia, Europe, and North and South America were covered by thick ice sheets and could not support trees or any plants at all (Graham, 1993; Hewitt, 2000; Imbrie & Palmer‐Imbrie, 1979; Milne & Abbott, 2002; Tiffney, 1985; Wen, 1999). During the cold phases and the advance of huge, deadly ice sheets, trees and many other organisms became extinct, but others survived in warmer regions of lower latitudes, termed refugia (e.g. Bennett et al., 1991; Comes & Kadereit, 1998; Delcourt & Delcourt, 1993; Hewitt, 2000; Milne & Abbott, 2002). Some refugia in Europe were relatively northern and inland (e.g. the northern Balkans), where their organisms were exposed to very low winter temperatures that should have had a strong influence on both plant and herbivore extinction or survival. Some European refugia were more southern and climatically milder (e.g. the Iberian Peninsula and southern Italy; Bennett et al., 1991; Provan & Bennett, 2008; Willis & van Andel, 2004). In Europe, northern refugia for broadleaf trees during Pleistocene glaciations are known for a small number of deciduous species, none of which have red autumn leaves: *Alnus glutinosa* (a nitrogen fixer with green autumn leaves), *Betula pendula* (yellow), *Fagus sylvatica* (yellow), *Fraxinus excelsior* (green and sometimes yellow), *Salix* sp. (yellow), *Corylus avellana* (yellow) and *Frangula alnus* (yellow) (Bhagwat & Willis, 2008).

The various reasons for the current differences in the global distribution of red *versus* yellow autumn leaves in trees, shrubs and climbers of the temperate and boreal regions lie, among other factors, in the geological history of the world in the Cretaceous, Tertiary and Quaternary, the outcome of plate tectonics that determined the directions of mountain ridges at the continental level (see below). By this, plate tectonics indirectly determined the climatic patterns and climate‐dependent extinction, survival, recolonization and adaptation throughout the Pleistocene, with its alternating waves of glaciation and warming.

In eastern North America, in western North and South America and in east Asia, the direction of the mountain ridges is from north to south (Hewitt, 2000; Milne & Abbott, 2002; Soltis et al., 2006; Tiffney, 1985), the outcome of the directions of continental *versus* ocean floor movements. By contrast, in Europe, the Alps and their eastward extensions form an east–west ridge (Milne, 2004; Milne & Abbott, 2002; Soltis et al., 2006; Tiffney, 1985). Accordingly, in North America and east Asia, when the waves of southward‐advancing ice during Pleistocene phases of glaciation influenced the biota, tree species and their specific and non‐specific insect herbivores could migrate to the warmer south in the valleys between the mountains, or along the ridges, according to their specific ecology, and *vice versa* following periods of warming and ice retreat. It resulted in the preservation of many ancient Tertiary floral and faunal elements there. Naturally, the directions of glaciations and glacial retreats, and of plant and animal migrations were not only opposite in South America but also along a north–south trail. In Europe, however, during the repeated drastic climatic changes in the Pleistocene, the biota was trapped again and again between the advancing ice from the north on the one hand and ice from the Alps and their eastern extensions from the south on the other (Imbrie & Palmer‐Imbrie, 1979). Thus, a larger proportion of the European species assembly (both plants and animals) became extinct, leaving a much smaller number of species that later spread from several refugia during warmer periods to re‐occupy landscapes freed from the ice cover (Comes & Kadereit, 1998; Milne, 2004; Milne & Abbott, 2002; Soltis et al., 2006; Tiffney, 1985). The great differences in extinction rates between Europe and other continents can be seen in the much smaller number of European deciduous tree species compared with those between eastern North America and east Asia (e.g. Lee et al., 2003; Milne & Abbott, 2002). Many more Tertiary elements are therefore found in North America and east Asia than in Europe (Milne & Abbott, 2002; Tiffney, 1985). These changes should be considered when discussing adaptations and geography of deciduous woody temperate and boreal species. Ignoring the impact of these dramatic changes concerning the current geography, diversity, physiology, herbivory and pathology of temperate/boreal species may lead to considerable mistakes. I demonstrate the differences in extinction between Europe, east Asia and North America with data about two genera with species expressing either yellow or red autumn leaves: *Quercus* and *Acer*. *Quercus* has about 240 species in North America, 100 in Asia and 22 in Europe, and *Acer* has about 100 species in east Asia, 10 in Europe and dozens in North America (many of them known only as fossils from the Tertiary).

Autumn leaf colouration usually predates leaf fall by days or weeks. It occurs not only in four basic colour types (yellow, red, brown and green) but also in combinations of these colours within taxa, within individual plants and even within individual leaves (3, 4, 5, 6). Yellow‐senescing leaves may also appear in temperate deciduous trees within weeks or even months before autumn because of severe drought (Addicott, 1982; Kramer & Kozlowski, 1979). The non‐green autumn leaf colours are the outcome of several distinct biochemical processes. Yellow autumn leaves are the outcome of degradation of chlorophylls and unmasking of pre‐existing carotenoids and xanthophylls. Red autumn leaves are the outcome of a combination of degradation of chlorophylls and autumnal production of anthocyanins (Lee, 2002; Matile, 2000). Brown autumn leaves are the outcome of degradation of chlorophylls, carotenoids and xanthophylls. Shedding of green autumn leaves may in many species reflect a superior adaptation of nitrogen supply, which in many of these species is the outcome of nitrogen‐fixing symbiotic bacteria (Archetti, 2009a; Renner & Zohner, 2022), although it is not always so. However, a certain small number of woody species have yellow or red autumn leaves as the outcome of other pigment types (Lee, 2007).

**FIGURE 3:**
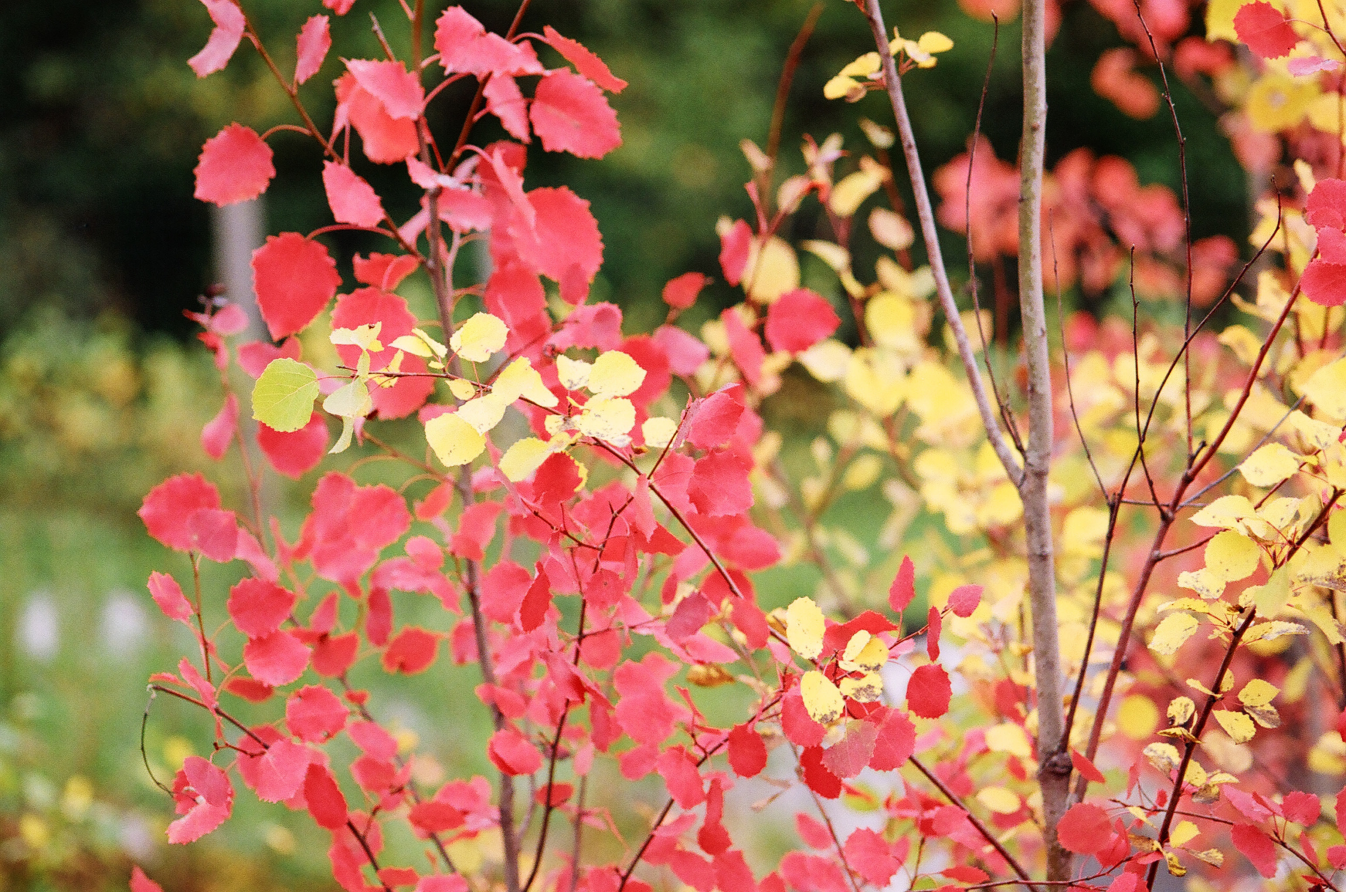
Two *Populus* sp. clones, one with red autumn leaves and the other with yellow ones, botanical garden, University of East Finland, Kuopio, Finland.

**FIGURE 4:**
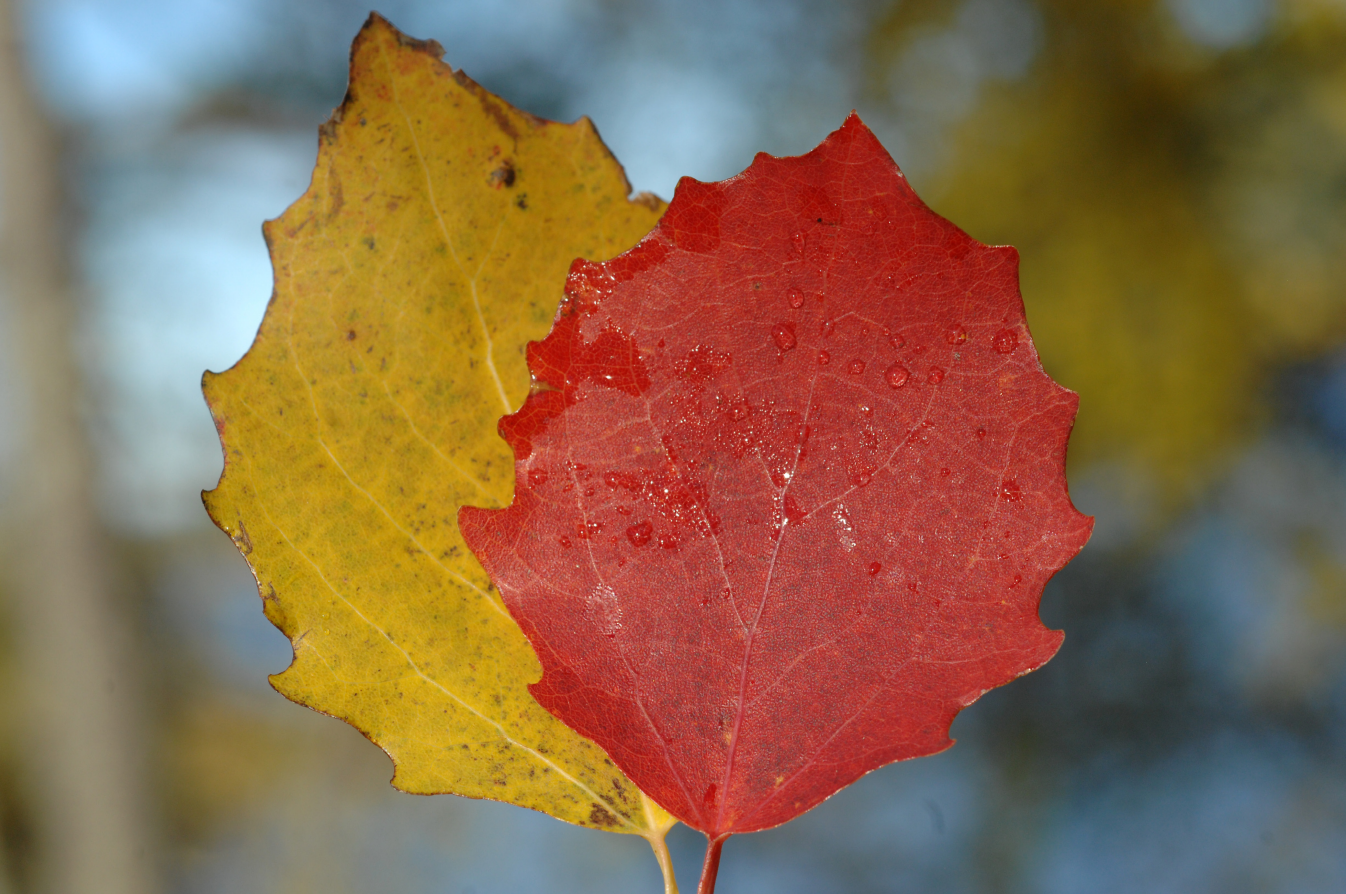
Two autumn leaves from the same *Populus* sp. trees, one red and the other yellow, Kuopio, Finland

**FIGURE 5:**
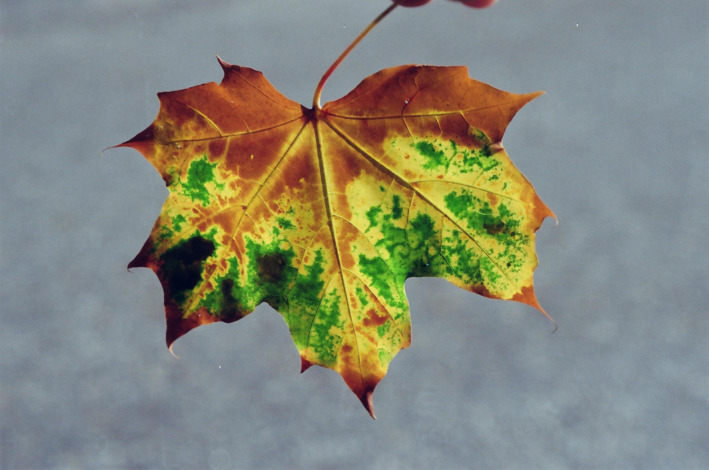
Multicolour *Acer* sp. leaf in the process of autumn leaf Colouration, Kuopio, Finland.

**FIGURE 6:**
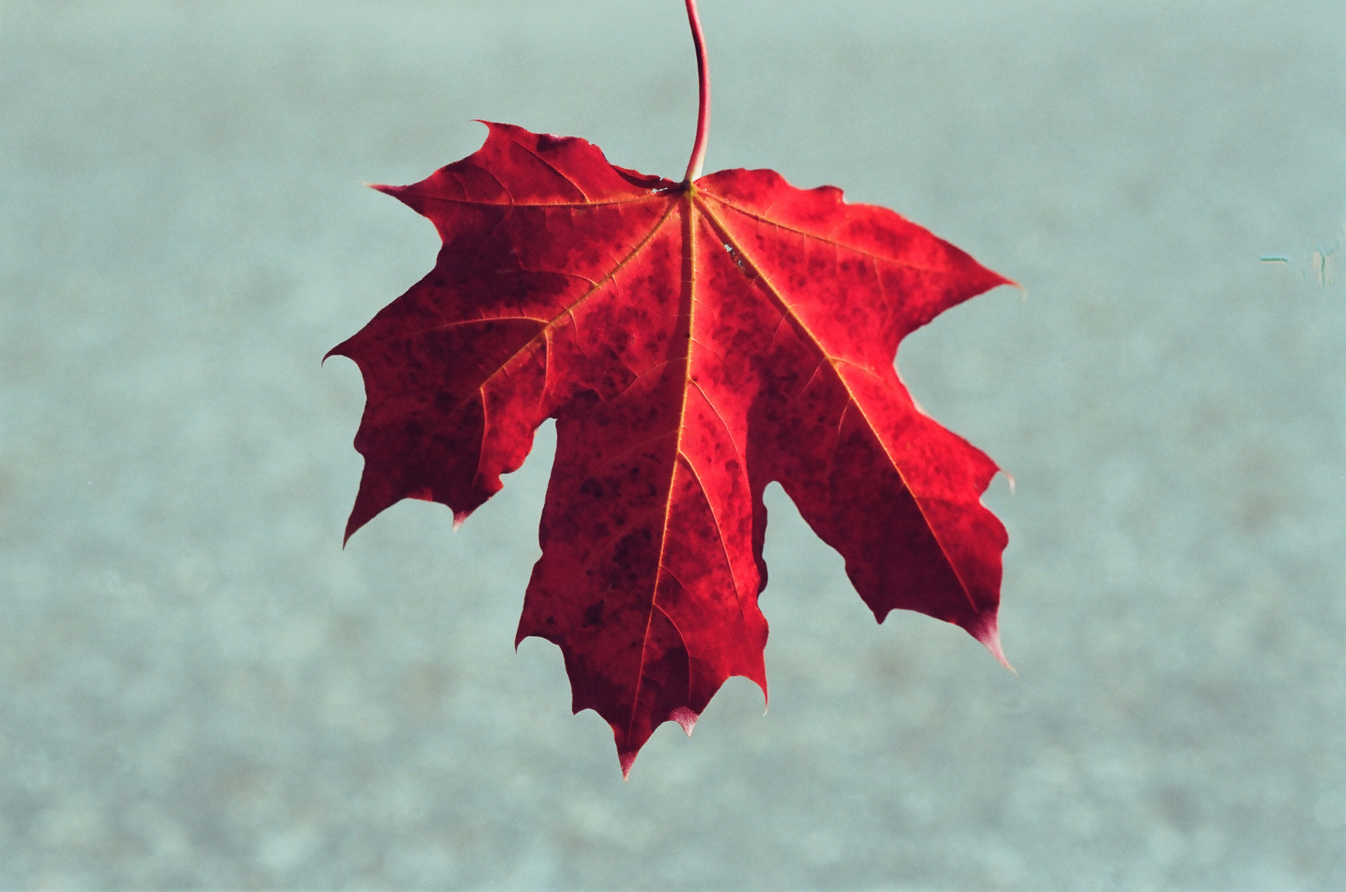
A red *Acer* sp. autumn leaf from the same tree of the leaf in Figure 5, Kuopio, Finland

Enhanced recovery of foliar nitrogen and other resources by their retranslocation to the branches and trunk is attributed to the protection from photoinhibition and photooxidation by anthocyanins (Archetti et al., 2009a; Close & Beadle, 2003; Feild et al., 2001; Hoch et al., 2001, 2003; Lee, 2002; Lee & Gould, 2002; Matile, 2000; Ougham et al., 2005). One of the most important functions of anthocyanins is to protect the cells from reactive oxygen species (ROS), although, until recently, their various locations within the leaves and within the cells (dissolved in vacuoles) caused disagreements among physiologists, because severe oxidation stresses may initially be more common within the chloroplasts than in the vacuoles but may quickly shift to the vacuoles (see Hughes et al., 2022). The common ‘understanding’ of the situation is a much too simplified and misleading description of the cellular system (see later when I discuss Hughes et al., 2022). Scavenging reactive oxygen species and defence from photoinhibition were proposed as important physiological reasons for the common existence of young red leaves in the tropics and elsewhere (e.g. Gould, 2004). Although low temperatures may indeed induce leaf and stem reddening in many species (Close & Beadle, 2003; Hughes, 2011), red leaves are not associated only with low temperatures (i.e. the common occurrence of red young leaves in the tropics) (Coley & Barone, 1996; Dominy et al., 2002; Juniper, 1994; Lee & Collins, 2001; Richards, 1996), and low temperatures are not always associated with red leaves, for instance the dominance of yellow autumn leaves in the cold autumn of Scandinavia (Holopainen & Peltonen, 2002; Lev‐Yadun & Holopainen, 2009). As shown by Manetas's group, anthocyanins seem to act as an alternative (but not necessarily superior or even fully compensatory) photoprotective mechanism to upregulation of xanthophyll cycle pigments (e.g. Kytridis et al., 2008), a topic later substantiated by many, including Hughes et al. (2012). Moreover, red leaves may in many cases (including in deciduous species) reflect nitrogen deficiency (e.g. Carpenter et al., 2014; Hughes et al., 2022; Kytridis et al., 2008).

The phenomenon of yellow and red autumn leaves in woody plants of the temperate/boreal zones should not be considered as a biological character standing by itself but rather as an interesting part of a much larger arena of leaf colouration related to defence from both herbivores and pathogens, and to cell, tissue, leaf, organ and whole plant physiology. The data and hypotheses emerging from studying non‐autumnal types of leaf colouration facilitate the understanding of the complicated phenomenon of autumn leaf colours. There is an overlap in the physiological and defensive (anti‐herbivory and anti‐pathogenic) functions of colourful autumn leaves with many colourful/non‐green young leaves and with colourful leaf undersides and colourful leaf margins (e.g. Archetti, 2009b; Archetti et al., 2009a; Cooney et al., 2012; Hughes & Lev‐Yadun, 2015; Hughes & Smith, 2007; Lee, 2002; Lee & Gould, 2002; Lev‐Yadun, 2016; Schaefer & Ruxton, 2011). The level of this overlap is only partly known.

Although in very cold temperate regions, low spring or autumn temperatures seem to be among the factors that selected for red leaf colouration, it is obviously not so in the tropics, with their many red young leaves (e.g. Dominy et al., 2002; Juniper, 1994; Lee & Collins, 2001; Richards, 1996), or in the Mediterranean region (Israel) where evergreen woody species such as *Pistacia lentiscus*, *Ceratonia siliqua*, *Quercus calliprinos* and *Ricinus communis* produce many red young leaves not only during the cooler winter and spring but also during the hot and dry Mediterranean summer, under night temperatures of at least 23–28°C and day temperatures of 30°C and more. In the tropics, red young leaves are part of a very common phenomenon of delayed greening that has been proposed to be associated with low nutritive value of these young leaves as defence from herbivory (e.g. Chen & Huang, 2013; Coley & Barone, 1996; Kursar & Coley, 1992), and in undermining the visual camouflage of invertebrate herbivores (Lev‐Yadun et al., 2004). In central Japan, the evergreen tree *Elaeocarpus sylvestris* has red senescing leaves all year‐round (Mogi et al., 2000), indicating again that low temperatures are not the sole factor that selects for red leaves. Moreover, in many floras, petioles and major veins of many herbaceous and woody species are red in many species in which the other parts of the leaf are green.

Another theoretical and practical issue related to autumn leaf colouration is the issue of leaf colour variability. This variability may be the outcome of variability in pigmentation among taxa and individuals, or position in the canopy of an individual tree, and may also be strongly influenced by exposure to sun irradiation, temperature, nutrition, reproduction, water or nutrient shortage, herbivore damage, and disease. This variability may also include differences in the timing of colour changes as regulated by genetics, as well as by abiotic and biotic environmental conditions (e.g. Lee, 2012; Sinkkonen et al., 2012). Several authors proposed that such variability in timing and intensity of autumn leaf colouration has been selected for by either physiological or anti‐herbivory reasons (e.g. Anderson & Ryser, 2015; Archetti, 2000; Archetti & Brown, 2004; Hagen et al., 2003; Hamilton & Brown, 2001; Ougham et al., 2005; Sinkkonen, 2006a, 2008; Sinkkonen et al., 2012). The results of Hagen et al. (2003) in *Betula pubescens* ssp. *czerepanovii* trees that turn yellow early in autumn (September) were negatively correlated with insect damage in the following growth season and did not match the results of Isaksen and Folstad (2014) for the same subspecies but in different years. Isaksen and Folstad (2014) found no difference in the attacks of the most common caterpillar species, *Epirrita autumnata*, on leaves, or in the oviposition by its mature females on twigs senescing early or late, and rejected the aposematic function of leaf colour in that case. Thus, it seems that at least for that specific combination of tree and insect species, the difference in susceptibility to herbivory is not only partly related to variability in phenology but is also year‐ and habitat‐dependent. However, at least for various Finnish *Betula pendula* (silver birch) genotypes, Sinkkonen et al. (2012) showed that the timing of autumn leaf colouration varies significantly among genotypes, and it was therefore proposed that this bears on fitness and evolution (Lev‐Yadun & Keasar, 2012). In that species, genotypes that expressed the strongest leaf reflectance in early autumn harboured more egg‐laying females of the specialist aphid *Euceraphis betulae* than those expressing strong leaf reflectance several weeks later. Interestingly, in Norway maple (*Acer platanoides*), reddish leaf colours were more frequent in partially dead trees than in healthy ones (Sinkkonen, 2008), an indication of additional parameters that may influence the variability in autumnal leaf colouration and not only the strength of chemical defence as proposed by the Hamilton group (e.g. Archetti, 2000; Archetti & Brown, 2004; Hamilton & Brown, 2001). Silfver et al. (2015) studied the phenology of shoot growth termination in various Finnish *Betula pendula* genotypes that differ in that character. Genotypes that terminated shoot growth earlier suffered from higher aphid egg loads after a short, but not after a long, autumn. The Sinkkonen et al. (2012) and Silfver et al. (2015) studies illuminated the need to document the intraspecific variability in autumn leaf colouration and the corresponding aphid attacks in many other taxa and under various environmental conditions.

Because of the above, when only one or only a small number of specimens are inspected in a botanical garden for autumn leaf colours, for the variability in timing of leaf colour change, for the colour itself or for the duration of leaves on the tree, the data cannot reliably represent the actual situation of any species. Considering such data as valid for understanding physiology, ecology and evolution of a species or of many species and for various calculations (e.g. Panchen et al., 2014; Renner & Zohner, 2019, 2020; Zohner et al., 2017; Zohner & Renner, 2017) is a critical and misleading mistake.

## 2 PHYSIOLOGISTS *VERSUS* ECOLOGISTS

The debates between physiologists and ecologists emerged not only because of the complexity of the system, because the partners on both sides do not master the knowledge of the other side, but also because there is a great difference in the scientific education and way of thinking and collecting data between plant physiologists and plant ecologists (I was trained, conducted research and thought for decades in both areas). Plant physiologists are trained to conduct experiments in which they commonly isolate and test one or just a few components out of many in the organismic or abiotic systems, usually in the laboratory, under controlled artificial conditions in growth chambers, or in non‐organismic and even in non‐cellular contexts. The very powerful practices of reductionism that allow precise molecular‐, chemical‐ or physical‐level measurements produce in certain cases results that are very much out of the actual biological context. This tendency has been increasing in recent decades and leads to many types of experimental mistakes and misunderstandings. In this connotation, I recall my postdoc experience, when I told my mentors that the protein product of the gene we were studying is associated with microtubules only when the tubulin was isolated from the cytoplasm and polymerized *in vitro* but not in the spindle when the cells were intact, and that it was thus not the microtubule‐associated protein in wheat (*Triticum*) that they were looking for. Indeed, it was found later to be a heat‐shock protein. When it comes to forest or landscape ecology of multispecies and multitrophic ecologies, as it is concerning the testing of questions such as the ecology and evolution of autumn leaf colouration, the way of thinking, and what and how things are measured by typical plant physiologists are in certain cases either partly or fully irrelevant, that is out of context (see later). For instance, even if within each cell or leaf, anthocyanins seem at first to be located in non‐optimal layers to protect from UV or from excess light (something that is not agreed on), the situation within the individual tree canopy, which in many cases is the unit of evolution, can be totally different, because outer canopy leaf layers may shade and defend with their anthocyanins the many inner or lower leaves, especially in species belonging to the late‐successional multilayered canopies (e.g. Horn, 1971). Such defence on the lower parts of the canopy by upper parts was demonstrated in the Saharan desert shrub *Retama raetam*, common in Israel, in which the upper branches shade the lower ones during the highly sun‐irradiated hot and dry summer (Mittler et al., 2001). Whole canopy physiology of mature trees is not easy to measure and indeed is not measured by most plant physiologists who study anthocyanins. In addition, some plant physiologists (e.g. Manetas, 2006) have proposed that anthocyanins may not be effective as sunscreens because there are other molecules that do it better. This was an early attempt to understand the issue, and very soon after Manetas changed this view (Karageorgou & Manetas, 2006; Kyparissis et al., 2007; Kytridis et al., 2008; Liakopoulos et al., 2006). Anthocyanins are expressed in leaves for several simultaneous functions (e.g. Gould, 2004), and what determines the evolution of their expression is the integral of their various gains and costs and not only specific single functions.

There are plant physiologists who do not consider the biosynthesis of anthocyanins and their sunscreen ability, that is photoprotection, as their major function in spite of their well‐known contribution in photoprotection in both young and senescent red leaves. Indeed, in many cases (30%, e.g. Gould et al., 2018), no photoprotective function was found. However, it is also known that there is a common confusion between photoprotection, that is protection from sunlight that causes photoinhibition and oxidative stress, and actual photoinhibition, that is reduction of CO_2_ assimilation. Many researchers test the photoprotective role of anthocyanins by testing their sunscreen effect, and this is not always sufficient to measure photoinhibition that requires measuring gas exchange and the activity of Calvin enzymes. Downregulation of Calvin enzymes can also induce photoinhibition, for instance at low temperatures. Photoinhibition is caused by excessive light on the one hand and triose and later sugar accumulation on the other hand (see Agati et al., 2021). Anthocyanins can act both as a sunscreen and as a carbon sink for these sugars. It should also be remembered that there are many young red leaves that still do not produce much (e.g. Kursar & Coley, 1992), which do not seem to have a need for a solution for excess sugar. Moreover, many senescing red leaves also do not produce much but rather serve as sources of materials for recycling, and there is no need for a sucrose sink that will enable better photosynthesis in such leaves. The need for a carbon sink is a real issue in leaves in the prime of their photosynthetic activity, but it should not be confused with the situation in both young and still developing leaves, and with senescing leaves when they are deep in the process of disintegration of their photosynthetic apparatus and in transport for recycling. Altogether, from the physiological point of view, anthocyanins can act as sunscreens, as scavengers of oxygen reagents and as a carbon sink. The question is when in leaf ontogeny, and where and when at cell, leaf and canopy levels, they do it. The answer to this question is not simple and not single even when dealing with an individual tree, and has never been studied in enough detail in any of the species that have autumn or non‐autumn red leaves. Anthocyanins are, however, powerful antioxidant compounds not only directly within cells but also because of their sunscreen ability at canopy level that limits light access to chloroplasts of shaded leaves, thus indirectly reducing ROS generation by the imbalanced photosynthetic machinery, especially under low temperatures.

Ecologists commonly measure things in much cruder ways than physiologists. Instead of measuring chemical and physical characteristics of specific molecules, changes in their amounts or specific metabolic responses, ecologists measure species abundance, numbers, interactions among whole organisms, etc., parameters that are much less delicate than individual molecules and the like. Therefore, ecology may look less precise but can be not less predictive than modern molecular physiology. This essay is not aimed to solve the potential conflicts between physiology and ecology, but just to illuminate the differences, something that can with time help understanding and likely solve the debates.

## 3 DEFENCE OF ANY TYPE IS IMPERFECT AND RELATIVE

Concerning all anti‐herbivory defences, it should be clear that no plant defence system is perfect, an issue already stated long ago (Crawley, 1983; Janzen, 1979; Lev‐Yadun, 2016; Rothschild, 1972). Assuming that a perfect defence system could exist is naïve. All types of defences probably have a cost, although the cost can be partially mitigated by defensive multifunctionality of some of the defences and following simultaneous types of non‐defensive (physiological, structural, storage and reproduction) gains by these characters. An organism has to pass through an evolutionary filter and make physiological, developmental and ecological decisions on how much defence can be achieved with the existing resources at any point in time and life cycle (Coley et al., 1985; Herms & Mattson, 1992). This implies that various defences must be age‐, cell type‐, organ‐, season‐ and ecology‐dependent (see Ochoa‐López et al., 2015; Ronel et al., 2009; Ronel & Lev‐Yadun, 2012) and that the defence has to change with plant ontogeny as a whole, according to organ age and condition (Barton & Koricheva, 2010; Boege & Marquis, 2005; Lev‐Yadun & Ne'eman, 2006), and also according to organ importance for fitness (e.g. Ronel & Lev‐Yadun, 2012). Moreover, the actual strength of defence also depends on the relative strength of the neighbours' defence (e.g. Atsatt & O'Dowd, 1976). Indeed, the relative strength of a plant's defence against herbivores as expressed in relation to the defence of its neighbours was experimentally demonstrated in various environments (e.g. Barbosa et al., 2009; Bergvall et al., 2006). Such relativity in plant defence by colourful autumn leaves was only partly tested experimentally or discussed concerning defence and only a few times, for example Archetti (2000) and Archetti et al. (2009a) concerning yellow *versus* red autumn leaves, Sinkkonen et al. (2012) concerning the timing of autumnal leaf colour change and Baisden et al. (2018) concerning various shades of red, and much remains to be done. The principle of being non‐perfect is also true for many aspects of plant physiology and for other characters that serve more than one function. Anthocyanins seem to be perfect candidates for such imperfection.

## 4 COLOUR‐BASED VISUAL LEAF DEFENCES

The conspicuous autumn leaf colouration phenomenon is simultaneously involved not only in several physiological functions but also in several visually based and chemically based defences. Evaluation of the potential anti‐herbivory colouration of yellow and red autumn leaves should be done with the perspective of the whole broad spectrum of physiological and defensive plant colouration. Defensive plant colouration (camouflage, aposematism, various types of mimicry, undermining herbivorous insect camouflage, masquerade, dazzle effects, trickery colouration and exploiting animals' perceptual biases) and related visual aspects had received very limited attention till the year 2000. Earlier important works are Hinton (1973), Wiens (1978), Rothschild (1986), Smith (1986) and Givnish (1990). A critical and clear note about this void in botany was given by Harper (1977, page 416) in his seminal book about plant population biology:

botanists have been reluctant to accept precisions of adaptations that are commonplace to zoologists and often seem reluctant to see the animal as a powerful selective force in plant evolution except in the curiously acceptable realm of adaptation to pollination! It may be that much of the fantastic variation in leaf form, variegation, dissection and marking that is known in the plant kingdom is accounted for by the selective advantage to the plant of associating unpalatability with a visual symbol.

Defensive plant colouration gained much more attention in the 21^st^ century (e.g. Archetti, 2000, 2009b; Archetti et al., 2009a; Burns, 2010; Cooney et al., 2012; Fadzly et al., 2009; Farmer, 2014; Hughes & Lev‐Yadun, 2015; Lev‐Yadun, 2001, 2009a, 2016, 2021; Lev‐Yadun et al., 2004; Lev‐Yadun & Gould, 2007, 2009; Lev‐Yadun & Holopainen, 2009; Lev‐Yadun & Ne'eman, 2012, 2013; Maskato et al., 2014; Niu et al., 2018; Quicke, 2017; Ruxton et al., 2004, 2018; Schaefer & Ruxton, 2009, 2011). Altogether, yellow autumn leaf colouration and red autumn leaf colouration are just two related phenomena out of many cases and mechanisms of anti‐herbivory plant colouration.

The major specific hypotheses about anti‐herbivory functions of non‐green leaf colouration are as follows: (1) Many herbivorous insects lack red receptors, thus seeing red leaves as dark objects rather than bright. It has thus been suggested that red foliage may hide trees from herbivores; that is, both young and old red leaves mimic dead ones or just look unlike green ones (Döring et al., 2009; Juniper, 1994; Karageorgou et al., 2008; Karageorgou & Manetas, 2006; Schaefer & Wilkinson, 2004; Stone, 1979). (2) Plants camouflage themselves (Klooster et al., 2009; Lev‐Yadun, 2006a, 2016, 2021; Niu et al., 2018). (3) Colourful young leaves attract herbivores and divert them from the costlier older ones (Lüttge, 1997). (4) Leaves appear as if already infested or diseased (Lev‐Yadun, 2003a, 2016; Lev‐Yadun & Niemelä, 2017; Smith, 1986; Soltau et al., 2009). (5) Lev‐Yadun (2014a, 2016, 2019, 2021); Farmer (2014); Yamazaki and Lev‐Yadun (2015); Yager et al. (2016); Yamazaki (2016); and Claudel et al. (2019) proposed mimicry and masquerade of various types. (6) Lev‐Yadun (2014b, 2015, 2016) proposed that zebra‐like white leaf variegation may defend leaves and other plant organs from herbivory by dazzle effects. (7) Wiens (1978) mentioned a personal communication with C. Dodson that suggested that red young leaves in tropical plants may be aposematic but did not elaborate on this. Red and yellow poisonous autumn leaves were suggested to be aposematic in many cases, and Müllerian and Batesian mimicry rings were also suggested to occur (Archetti, 2009b; Archetti et al., 2009a; Lev‐Yadun, 2006b, 2009b, 2016, 2019, 2021; Lev‐Yadun & Gould, 2007, 2009). (8) Young leaves expressing delayed greening are less attractive to herbivores since they are less nutritious (Coley & Barone, 1996; Kursar & Coley, 1991, 1992). (9) Red autumn leaves (and until the year 2009 also yellow) signal to insects that the trees are well defended according to Zahavi's handicap principle, commonly known as the co‐evolutionary hypothesis (Archetti, 2000; Archetti et al., 2009a; Archetti & Brown, 2004; Hamilton & Brown, 2001). (10) Mature leaves signal *via* red colour that they are a nutrient‐poor resource (Sinkkonen, 2006a, 2006b, 2008). (11) Red autumn leaves function according to the ‘defence indication hypothesis’ (Schaefer & Rolshausen, 2006a), stating that red leaves reliably signal that they are chemically defended because anthocyanins and various chemical plant defences originate from the same metabolic branch. This understanding originated in Fineblum and Rausher (1997) concerning defensive flower colours. This hypothesis predicted that fewer herbivorous insects [and Lev‐Yadun & Gould, 2007 suggested any herbivore taxon sensitive to these metabolites] would feed on vegetative parts of plants that have strong anthocyanin colouration, because in many cases, it correlates with the strength of a chemical defence. (12) Red leaf margins can serve as an aposematic signal in toxic leaves (Cooney et al., 2012; Hughes & Lev‐Yadun, 2015) and undermine insect camouflage (Hughes & Lev‐Yadun, 2015), or when the colourful margins are not continuous, they may serve as an indication of previous damage that upregulated defensive mechanisms when it was partly eaten (Hughes & Lev‐Yadun, 2015). Hypotheses 8–12 are also types of aposematism. (13) Leaf colouration is implicated in tritrophic interactions because the colouration undermines herbivorous insect camouflage, allowing better visual detection of the herbivorous insects by their predators or causing avoidance of such leaves by the herbivores (Lev‐Yadun, 2016, 2021; Lev‐Yadun et al., 2004; Lev‐Yadun & Gould, 2007). (14) Red leaves attract predaceous species whose presence reduces the total herbivore load (Yamazaki, 2008a, 2008b). (15) Red and yellow autumn leaves signal to herbivores that the leaves are going to be shed soon (Lev‐Yadun & Gould, 2007). (16) Red anthocyanins in autumn leaves may mask the attractive yellow colouration, and by this, they attract fewer aphids (Döring et al., 2009). (17) When colourful margins are not continuous because leaf margins were consumed, they may serve as an indication to herbivores and to their predators of previous damage, which among other influences upregulated defensive mechanisms when it was partly eaten (Hughes & Lev‐Yadun, 2015). (18) Regarding the origin of red autumn leaves, it has been postulated that red autumn leaves are in many cases an ancient Tertiary adaptation to currently partially extinct herbivore fauna (Lev‐Yadun & Holopainen, 2009). Many of the above suggested visual defences are relevant to the discussions about the evolution of yellow and red autumn leaves. Partly or fully ignoring them may lead to misunderstandings of the evolution of autumn leaf colouration and to various mistakes.

## 5 RED LEAVES ARE PROBABLY BETTER DEFENDED FROM HERBIVORY AND FUNGAL ATTACKS THAN GREEN AND YELLOW ONES

The fact that red leaves, in all seasons and at all stages of leaf ontogeny, are less attractive for herbivorous insects than green ones is well documented (e.g. Baisden et al., 2018; Gong et al., 2020; Karageorgou et al., 2008; Karageorgou & Manetas, 2006; Maskato et al., 2014; Numata et al., 2004; Prokopy et al., 1983). Concerning the related phenomenon of red fruit, it is an issue with specific complications, because there are unripe red fruits in a number of taxa that were proposed to visually signal about still being non‐edible or even toxic (Lev‐Yadun, Ne'eman, & Izhaki, 2009a), and other fruits may be ripe and sweet when red, signalling edibility to seed dispersers (van der Pijl, 1982). From what we see in fruits, it is tempting to suggest that in certain cases red may not signal something specific but just signal ‘pay attention’. Interestingly, in a comparison of mechanical defences in 36 woody species with red young leaves, with 40 woody species with green young leaves, Chen and Huang (2013) found that species with red young leaves had a much weaker mechanical defence, indicating that the red ones are better defended chemically or by other means, findings supported by earlier findings of actual lower herbivory on young red leaves of *Quercus coccifera* (Karageorgou et al., 2008; Karageorgou & Manetas, 2006), and by later chemical (Maskato et al., 2014) and structural findings for many more Chinese species in five different forests (Chen et al., 2021).

The actual defensive function of delayed greening, when in many species the young leaves are red, was tested in the Pasoh Forest Reserve in a typical lowland forest of Peninsular Malaysia by Numata et al. (2004). They showed that seedlings of various species of the genus *Shorea* (Dipterocarpaceae), which express delayed greening, indeed suffered less damage from insect herbivory than species with regular greening. Another test of the possibility of the defensive potential of delayed greening and red young leaves was conducted in Ecuador and Panama and also showed that this character is associated with reduced herbivory (Queenborough et al., 2013). Gong et al. (2020) tested the roles of red young leaves in tropical trees and found that defence from insect herbivory rather than photoprotection explains this phenomenon. Similarly, in Heishiding nature reserve, located in southern China, it defended seedlings from herbivory in dry sites but not in wet ones (Li et al., 2021).

Gerchman et al. (2012) found that the conspicuous purple tufts of leaves (‘flags’), which often terminate erect inflorescences in the Mediterranean annual *Salvia viridis* and attract insect pollinators to the flowering plants, have in addition an aposematic potential towards herbivores. Maskato et al. (2014) studied in more detail the finding by Gerchman et al. (2012) and found that locusts preferred green cabbage leaves over anthocyanin‐rich red cabbage leaves. They also found that female *Pieris* butterflies avoid laying eggs on anthocyanin‐rich red foliage and that larvae feeding on red cabbage leaves exhibited significantly lower growth rates and a longer duration of larval development. Maskato et al. (2014) thus proposed that from an evolutionary perspective, red foliage colour may serve as an honest defensive cue, and I say that in many cases, it is probably even a signal.

A recent paper (Baisden et al., 2018) that compared horticultural varieties of ten common woody species with colourful leaves to their varieties that have green leaves found that cultivars with red, blue or purple leaves suffered less from insect feeding and were occupied by fewer caterpillars. This was also true for cultivars with colourful autumn leaves, although the effect was not as strong as in cultivars expressing red, blue, or purple leaves throughout the growing season. The possibility of the simultaneous operation of olfactory aposematism *via* volatiles produced by the same biochemical pathway that produces the pigments has not been tested in any of these cases.

## 6 VISUAL APOSEMATISM

Aposematic (warning) colouration is a biological phenomenon in which poisonous, dangerous or otherwise unpalatable or unprofitable organisms visually advertise these qualities to animals (Cott, 1940; Edmunds, 1974; Ruxton et al., 2004, 2018) as defence from predation. The evolution of aposematic colouration or other types of aposematic signalling is based on the ability of target enemies to associate the visual, chemical or other signals with risk, damage or non‐profitable handling, and later to avoid such organisms as prey (Cott, 1940; Edmunds, 1974; Ruxton et al., 2004). In certain cases, there is even an innate tendency to avoid objects with certain colours or colour patterns (Lindström et al., 1999; Ruxton et al., 2004). Typical colours of aposematic animals are yellow, orange, red, purple, black, white, brown and combinations of these (Cott, 1940; Edmunds, 1974; Ruxton et al., 2004, 2018). The common defence achieved by aposematic colouration resulted in the evolution of many mimicking animals. The mimics belong to two general categories, Müllerian mimicry (defended ones look similar) and Batesian mimicry (non‐defended ones look like defended ones) (e.g. Ruxton et al., 2004; Wickler, 1968), although there are intermediate situations known as quasi‐Batesian mimicry (Rowland et al., 2010; Speed, 1999).

Aposematic (warning) colouration seems to be a common defence in plants, although it received very limited and mostly sporadic attention till the year 2001. As in animals, aposematic colouration in plants is commonly yellow, orange, red, brown, black, white or combinations of these colours (Lev‐Yadun, 2001, 2016). Aposematic colouration is expressed in many thorny, spiny, prickly and poisonous plants, and in plants that are unpalatable or of low nutritive value for various other reasons, as well as in plants that are unsuitable habitats for small herbivores because of their colour or texture (Fadzly et al., 2009; Lev‐Yadun, 2001, 2009a, 2016, 2021; Rothschild, 1986). Moreover, plants can be aposematic as the outcome of defences acquired directly or indirectly from other organisms, including plants, fungi, bacteria, insects and possibly also various other invertebrates and vertebrates (Halpern et al., 2007; Himanen et al., 2010; Lev‐Yadun, 2009a, 2016; Lev‐Yadun & Halpern, 2007; Rothschild, 1972). Like in animals, plants that mimic aposematic plants or aposematic animals are also classified as either Müllerian or Batesian mimics (Lev‐Yadun, 2003a, 2003b, 2009b, 2016, 2019, 2021; Lev‐Yadun & Inbar, 2002; Yager et al., 2016). Many types of aposematic plant colouration simultaneously serve other functions such as physiological, communicative and even other defensive functions (Archetti et al., 2009a; Gould, 2004; Lev‐Yadun, 2003a, 2016; Lev‐Yadun & Gould, 2007, 2009; Shelef et al., 2019). It is therefore difficult in many cases to evaluate the relative functional share of visual aposematism *versus* other functions in various plant colour patterns and to identify the specific selective agents involved in the evolution of their colouration (Lev‐Yadun, 2009a, 2016).

It should be remembered that for the vast majority of species of all animal and plant taxa proposed or posited to be aposematic, aposematism has never been proved by showing avoidance learning or genetically based avoidance! Still, proposed aposematism is an excellent research tool that explains various interactions among plants and herbivores and the existence of many characters in many plant taxa (e.g. Lev‐Yadun, 2016). Moreover, the concept of aposematism, even when not proved yet for certain taxa, may stimulate further research.

## 7 OLFACTORY APOSEMATISM OF COLOURFUL AUTUMN LEAVES

Olfactory plant aposematism is the phenomenon whereby defended plants, usually toxic ones, signal their defence by volatiles in order to deter mammalian or insect herbivores (e.g. Eisner & Grant, 1981; Karban, 2015; Launchbaugh & Provenza, 1993; Lev‐Yadun, 2014c; Lev‐Yadun, Ne'eman, & Shanas, 2009b; Massei et al., 2007; Provenza et al., 2000). The visual anti‐herbivory signalling of colourful autumn leaves is probably supplemented in many species by an olfactory one (e.g. Blande et al., 2010; Holopainen, 2008; Holopainen et al., 2010), but that issue has not been studied yet on a broad scale and deserves much more research attention. The fact that there are good physiological measurements of significant volatile release from yellow autumn leaves (e.g. Keskitalo et al., 2005) supports the possibility of the simultaneous operation of olfactory along with visual aposematism (or other visual leaf defences) by these leaves. Holopainen et al. (2010) found that leaves of *Betula pendula* that are about to be shed emit *Cis*‐3‐hexenol, an indicator of cellular disintegration, which becomes a dominant volatile just before leaf abscission. This, combined with the yellow colour that cues or signals that the leaves are going to be shed soon (e.g. Lev‐Yadun & Gould, 2007), may at least cue to aphids that the leaves are close to shedding.

## 8 YELLOW AND RED AUTUMN LEAVES

The 1990s were the beginning of a period of a gradual better understanding of the physiological roles of leaf colours in general and of red leaves in particular. Improved technical abilities were not less important than theoretical understanding of the physiological aspects of leaf colour in general. Reviewing these physiological studies in depth is outside the scope of this review, but see Agati et al. (2021) and Hughes et al. (2022).

Only one non‐physiological hypothesis about the function of yellow and red autumn leaf colouration was formulated in the 20^th^ century (Stiles, 1982). Stiles's (1982) hypothesis that conspicuous autumn leaf colours in temperate forests may signal frugivorous birds about their ripe fruits (fruit flags) was not accepted by all (e.g. Burns & Dalen, 2002; Schaefer & Ruxton, 2011; Willson & Hoppes, 1986). Indeed, Stiles's (1982) hypothesis has several basic inherent problems. It does not explain why dioecious species have conspicuous autumn colours in leaves of male individuals that carry no fruit, and by this, it may divert potential seed dispersers from the fruit. Moreover, many female and monoecious trees with colourful autumn leaves already carry no fruit when the leaves become colourful, or they have wind‐dispersed seeds (Lee, 2002). However, if additional simultaneous physiological and defensive functions of such autumn leaf colours are considered, the fruit flag hypothesis may be correct, although certainly not an exclusive or a full explanation for the evolution of yellow and red autumn leaf colours, and in any case should be limited to the small number of species that carry ripe animal‐dispersed fruit in autumn. Moreover, since the colour contrast between the ripe fruits and their background is an important visual factor in fruit identification by birds that have an important role, especially in long‐distance seed dispersal (Burns et al., 2009; Cazetta et al., 2009), the species expressing colourful autumn leaves, especially yellow ones, which provide more contrast with red, dark blue or black fleshy fruits than red leaves, are better candidates for taking part in enhancing seed dispersal in certain species.

The case of fruit flags as proposed by Stiles's (1982) is typical of all potential functions of yellow and red autumn leaf colouration. This is true for both the functions that are marginal, such as fruit flags, and for the much more important ones, such as photoprotection and defence from herbivory or pathogens. In all types of proposed functions of yellow and red autumn leaves, there is not a single one that explains everything. Although it is clear from the literature that for certain scientists this multifunctionality may be hard to accept, this complicated and sometimes even confusing situation is one of the things that makes the study of autumn leaf colouration so fascinating.

## 9 STUDYING YELLOW AND RED AUTUMN LEAVES IN THE YEARS 2000–2009

Significant interest, along with hot debates about the functions and evolution of yellow and red autumn leaves, characterized the first decade of the 21^st^ century. Autumn leaf colours, which for many decades, were only infrequently studied or discussed, suddenly received lots of focused scientific attention (about 90 papers directly discussing it), and also attracted media attention. The fact that millions of tourists travel each autumn to view and enjoy landscapes with colourful autumn leaves, and the significant economy involved, added to the public and scientific interest in that phenomenon. There were and still are several parallel but theoretically different hot debates (see below) about the origin, evolution and functions of yellow and red autumn leaves among physiologists, among ecologists, and between physiologists and ecologists. Green and brown autumn leaves were almost ignored at that stage.

The first hot debate blazed between plant physiologists and plant ecologists: the sole role of various physiological functions of yellow and red autumn leaves *versus* the suggested roles of defence from herbivory in selecting for conspicuous autumn leaf colours. Some physiologists dismissed altogether the possibility of an anti‐herbivory role of yellow and red autumn leaf colouration, especially the co‐evolutionary hypothesis. The second debate stormed among plant physiologists concerning the various physiological roles of anthocyanins. The third was among ecologists. Some ecologists argued for some years that there is no proof of any anti‐herbivory function of colourful autumn leaf colouration, and others posited correctly that in many cases, yellow autumn leaves attract aphids and do not repel them.

The 21^st^ century opened with two papers on the physiology of autumn leaves as influenced by anthocyanins: Matile (2000) and Hoch et al. (2001) on the de novo production of anthocyanins in senescing autumn leaves that will be shed soon as photoprotection, especially in cold habitats. Simultaneously with the re‐illumination of the long‐known but commonly ignored de novo production and functions of anthocyanins in red autumn leaves by the two above citations, two papers from the Oxford group of the excellent and highly creative evolutionist, Professor Bill Hamilton (Archetti, 2000; Hamilton & Brown, 2001), initiated the first and in a way still naïve generation of anti‐herbivory hypotheses of autumn leaf colouration, which was proposed to operate according to Zahavi's handicap signalling principle. The hypothesis formulated and published by the Hamilton school (Archetti, 2000; Archetti & Brown, 2004; Hamilton & Brown, 2001) was known as the ‘co‐evolutionary’ colourful autumn leaf hypothesis. They considered red and yellow autumn leaves together under the title of ‘conspicuous colours’ or ‘bright colours’, as indicating the strength of the chemical anti‐herbivory defensive potential of the leaves of trees with such bright autumn leaf colours. This defence was posited to be especially aimed towards aphids. Archetti (2000), in the first paper presenting that hypothesis, posited that these defensive leaf colours do not operate *via* visual aposematism. The special theoretical aspect of the co‐evolutionary hypothesis is that the potential reduced fitness of the attacking insects is not only of those that land and may feed on the trees in autumn, which understandably are either supposed to respond or not respond to the proposed tree's colourful visual defensive signalling. The signalling is also aimed at repelling autumnal aphids from laying eggs on the woody parts of the trees (not on the colourful autumn leaves), eggs that would hatch the following spring. If the trees are chemically well defended, the development of the next aphid generation hatched the following spring will be damaged (Archetti, 2000; Archetti & Brown, 2004; Hamilton & Brown, 2001). Archetti (2000) presented a game‐theoretic model incorporating the basic assumptions of the hypothesis about the signalling between the trees and their parasites in the form of Zahavi's handicap principle. Hamilton and Brown (2001) compiled published data about autumn leaf colour in 262 tree species and about the aphid species found on them (their findings will be discussed later in this essay). Archetti and Brown (2004) discussed in great detail the background and the essence of the co‐evolutionary hypothesis. Soon after, Brown (2005) described the early stages of development of the hypothesis by Hamilton and his students. It should be remembered that damage by aphids is difficult to evaluate, because unlike chewing caterpillars they leave no visual signs. It is thus different from caterpillars, gallers, leafminers and chlorophyll suckers. This lack of visual damage arises difficulty in testing the coevolutionary theory. The essential updated aspects of the co‐evolutionary hypothesis were recently given in Pena‐Novas and Archetti (2022): A negative correlation between red autumn leaves and aphid numbers/diversity is expected in an intraspecific comparison, that is fewer insects on individual trees with stronger red colour of their leaves. In an interspecific comparison, the co‐evolution hypothesis predicts a positive correlation: Tree species co‐evolving with more insect species are more likely to evolve red leaves in autumn. However, I suggest that the compiled data about the aphid diversity on the trees by Hamilton and Brown (2001) might be less relevant than the data on their actual overall damage, a type of data that were either not available or not compiled. My critique does not negate the co‐evolutionary hypothesis but rather points to the types of data needed in order to better test and improve it.

The first generation of the co‐evolutionary hypothesis (Archetti, 2000; Archetti & Brown, 2004; Hamilton & Brown, 2001) was very fruitful not only for its own sake but especially because it stimulated many and diverse theoretical discussions and empirical studies on both the physiological and anti‐herbivory mechanisms of red and yellow autumn leaves. In any case, I suggest that the fact that there are many species‐specific aphids on trees with red autumn leaves (Hamilton & Brown, 2001) is strong evidence of the antiquity of this adaptation.

Unfortunately, Hamilton passed away in the year 2000 from an illness, and we all lost the opportunity of seeing his expected additional contributions to understanding the functions and evolution of yellow and red autumn leaf colouration when the first generation of the co‐evolutionary hypothesis turned out to be much too simplistic. Two response papers (Holopainen & Peltonen, 2002; Wilkinson et al., 2002) explained that contrary to the suggestion by the Hamilton group, aphids are usually attracted to yellow leaves rather than repelled by them (see also Chittka & Döring, 2007). Holopainen and Peltonen (2002) suggested that leaves that have just turned yellow are a good indication to aphids of nitrogen availability in the form of free amino acids, an attracting cue rather than a repelling signal. Later, Holopainen et al. (2009) showed that various aspects of aphids' life history may in certain species influence their ability to exploit yellow leaves. Wilkinson et al. (2002) posited that rather than signalling defensive qualities to aphids, especially since many of them are drawn to yellow leaves, this colouration serves as a sunscreen (a physiological role), and that red colours help to warm leaves and also function as antioxidants.

Another debate stormed and is still storming among plant physiologists concerning the various physiological roles of anthocyanins in relation to their location within cells, tissues, organs, and the canopy, as well as concerning their role in protection from UV, as defence from excess of visible light, and the proposed role of dark red colour in warming the leaves in cold habitats (see Hughes et al., 2022; Lee, 2012). Concerning the possibility that warming the leaves in cold autumns is at least one of the reasons for the evolution of red autumn leaves, two facts seem to indicate that this is usually not so. The first is that yellow autumn leaves dominate the very cold landscapes of Scandinavia (Holopainen & Peltonen, 2002; Lev‐Yadun & Holopainen, 2009). The second is the fact that most woody species with colourful autumn leaves have yellow leaves (Archetti, 2009a). However, this does not negate the possibility that warming autumn leaves by being red helps in retrieving resources from the senescing leaves in specific cases.

Concerning the hypothesis that Zahavi's handicap principle operates in colourful autumn leaves (e.g. Archetti, 2000, 2007a, 2007b; Archetti & Brown, 2004, 2006; Archetti & Leather, 2005; Brown, 2005; Hagen et al., 2003, 2004; Hamilton & Brown, 2001), the idea was partly (Chittka & Döring, 2007; Holopainen & Peltonen, 2002; Lev‐Yadun, 2006b; Lev‐Yadun & Gould, 2007, 2009; Ougham et al., 2008; Ramirez et al., 2008; Sinkkonen, 2006a, 2006b; Wilkinson et al., 2002) or wholly (Hatier & Gould, 2008; Ougham et al., 2005; Rolshausen & Schaefer, 2007; Schaefer & Gould, 2007; Schaefer & Rolshausen, 2006a, 2007a; Schaefer & Wilkinson, 2004; Yamazaki, 2008a) discounted on various grounds. Lev‐Yadun (2006b) and Lev‐Yadun and Gould (2007, 2009) emphasized that the operation of aposematism in colourful autumn leaves does not exclude the possible simultaneous operation of any other types of visual or non‐visual defence, as well as providing various physiological gains (see also Hatier & Gould, 2008).

The opposition to the first generation of colourful autumn leaf co‐evolutionary handicap hypothesis by ecologists reflected or was the outcome of the complicated biological facts involved, that is the simultaneous operation of various and sometimes contrasting physiological and anti‐herbivory functions of yellow and red autumn leaf colouration. In addition, further complicating the situation, the various functions of autumn leaf colouration probably differ in their importance with time and with the ontogenetic colour changes even within a single leaf, let alone in a flora or a broad geographical region (see Lev‐Yadun & Gould, 2007; Ougham et al., 2008). In parallel, many physiologists were certain that physiological functions, especially photoprotection, explained everything.

At the same time that the third paper (Archetti & Brown, 2004) was published by the Hamilton group, Lev‐Yadun et al. (2004) suggested that among other defensive and physiological functions, many patterns of plant colouration, including yellow and red autumn leaves, may undermine the camouflage of small invertebrate herbivores (see also Lev‐Yadun, 2009a, 2016, 2021; Lev‐Yadun & Gould, 2007). Accordingly, the herbivorous invertebrates would be vulnerable to predation if they occupied leaves that do not match their colour, and in addition, they may avoid inhabiting plant surfaces with unsuitable colouration because of their fear of predation to the benefit of the plants. This hypothesis attempted to provide a non‐exclusive unifying general anti‐herbivory explanation for many of the non‐variegated vegetal colouration types found in leaves, barks, flowers and fruits, and in variegated plants, only when the colour patches are very much larger than the size of the herbivores (Lev‐Yadun, 2016, 2021). This hypothesis, however, like many others, does not contrast or exclude any other previous or future explanations of autumn leaf colouration. This hypothesis was mistakenly considered to operate *via* leaf variegation (Schaefer & Rolshausen, 2006a), and my repeated comments on this (Lev‐Yadun, 2006b, 2009a; Lev‐Yadun & Gould, 2007, 2009) did not help to fix it (see Schaefer & Ruxton, 2011, page 166).

Ougham et al. (2005) stressed (correctly) the importance of and the need for good documentation of the physiological role of autumn leaf colouration. They argued that the handicap anti‐herbivory signal proposed by the Hamilton group (Archetti, 2000; Archetti & Brown, 2004; Hamilton & Brown, 2001) is not costly, but Ougham et al. (2005) did not consider at that stage that unlike yellow, the de novo‐produced red pigments in autumn leaves (e.g. Hoch et al., 2001; Matile, 2000) are indeed costly, which, according to the most common views but not all (see Lachmann et al., 2001), is a basic feature of signals involved in the operation of Zahavi's handicap principle (Zahavi, 1975; Zahavi & Zahavi, 1997), including aposematism (Holen & Svennungsen, 2012). Thus, although the physiological aspects of autumn leaf colouration, especially of red, are certainly of significant importance, they do not negate various anti‐herbivory functions, including by visual signalling. As was later realized, anti‐herbivory olfactory signalling (e.g. Holopainen, 2008), and not only visual signalling, is also involved in an unknown number of species that express colourful autumn leaves.

Lev‐Yadun (2006b) posited that colourful autumn leaves of certain taxa may be aposematic because they are toxic for certain herbivores. Later, Lev‐Yadun and Gould (2007) suggested that both yellow and red autumn leaves may, in addition to their various physiological and other defensive functions, also serve as a visual warning that the leaves are going to be shed soon, which is reliable, honest and critical information about a significant risk to insect mortality. This is vital information for insects that need leaves that will remain on the tree for longer than several days, to use as a habitat, especially for those insects that after the leaves fall cannot climb back or fly back to the canopy from the ground. Leaf fall is a well‐known agent of insect mortality (Faeth et al., 1981), and various insects may refrain from occupying leaves that are soon to abscise (Glinwood & Pettersson, 2000; Karban, 2007). Thus, the signalling tree will incur less herbivory, and the insects that respond to the signalling may suffer less mortality. It is already known that certain aphid species will lay eggs on trees whose leaves have just started to change colour and are yellow‐orange, but they usually refrain from occupying trees with red leaves (Furuta, 1986). Thus, the potential for such plant–insect communication by yellow and red autumn leaf colouration was already known to exist for decades.

Lev‐Yadun and Gould (2007) reviewed the issue of yellow and red autumn leaf colouration and concluded that (true for the year 2007) these colours have at least six potential visual anti‐herbivory functions, in addition to what seemed then to be better measured and understood, that is several important physiological roles. The first proposed defensive function of these colours was that they serve as a signal that the trees are well defended (a case of Zahavi's handicap principle) (Archetti, 2000; Archetti & Brown, 2004; Archetti & Leather, 2005; Hagen et al., 2003, 2004; Hamilton & Brown, 2001). The second, related and overlapping hypothesis was that red and yellow autumn leaves of toxic species function as aposematic colouration (Lev‐Yadun, 2006b; Lev‐Yadun & Gould, 2007) and probably include both Müllerian and Batesian mimicry (Lev‐Yadun & Gould, 2007). The third was that they function according to the ‘defence indication hypothesis’, stating that red leaves are chemically defended because the biosynthesis of anthocyanins correlates with the biosynthesis of various defensive compounds (Schaefer & Rolshausen, 2006a), a hypothesis overlapping aposematism. The fourth was that it undermines herbivorous insect camouflage (Lev‐Yadun et al., 2004; Lev‐Yadun & Gould, 2007). The fifth hypothesis, a modification of the co‐evolutionary hypothesis and actually also aposematism, proposed by Sinkkonen (2006a, 2006b) held that yellow foliage signals to sucking herbivores that the tree is becoming of poor‐quality food. Accordingly, much of the within‐population variation in autumn leaf colours can be explained by differences in resource allocation of the trees to sexual reproduction; that is, reproductively active woody plants express early and strong autumn leaf colours in order to protect seeds and other reproductive tissues from pests that lay eggs in the autumn. The logic behind positing this hypothesis was as follows: (1) When many seeds develop, a woody plant allocates considerable nitrogen amounts to seeds. (2) If mature sucking insects feed and reproduce on such hosts, their flightless offspring that hatch after seed ripening will suffer from poor‐quality nutrition. (3) If insects exist there when the seeds develop, in order to forage on the nitrogen‐rich phloem veins that transport these precious resources to the developing seeds, this will damage the quality and quantity of developing seeds, reducing the tree's fitness. (4) If, on the contrary, insects are able to recognize reproductively active plants in the autumn and refrain from laying eggs on them, both the insects and the plants benefit. The flightless offspring of insects will thus feed on plants that provide more nitrogen and will be able to do it for longer periods than while infesting strongly reproducing plants. Altogether, the trees improve their reproduction by lowering the amounts of lost resources because of high pest loads. Sinkkonen (2006a) tested the reproductive insurance hypothesis in mountain birch (*Betula pubescens*) trees and found that the number of ripening female catkins influenced the timing of yellow autumn leaf colour expression; that is, the autumnal colour change was earlier when the tree produced plenty of female catkins. This also served as a supporting case for the co‐evolutionary hypothesis. Thus, the hypotheses and experiments concerning the timing of leaf senescence and autumnal colour change should also consider the reproductive effort. This subhypothesis should of course be tested in additional taxa and ecologies. Sinkkonen (2006b) supposed that although physiological factors were the origin of autumnal leaf colour changes, the visible colour cue utilized by insects has evolved several times to an honest signal that reveals the unsuitability of the potential host in the near future. Sinkkonen (2006b) suggested that his reproductive insurance hypothesis may help in understanding why bright autumn leaf colours are rare among herbaceous plants, a related phenomenon that has not received the theoretical attention it deserves. The sixth and last hypothesis proposed till the year 2007 was that conspicuous autumn leaf colours might deter insects from occupying the soon‐to‐be‐shed leaves (Lev‐Yadun & Gould, 2007).

Soon after, Yamazaki (2008a, 2008b) proposed another defensive hypothesis, suggesting that there are situations where yellow or red autumn leaf colours may signal about tree quality to myrmecophilous aphids that will attract aphid‐tending ants during the following spring. These ants, when occupying the trees, will defend the trees from other aphid species and from other herbivores. Yamazaki (2008a, 2008b) posited that it is necessary to examine colour vision in the relevant hemipterans in order to support his hypothesis.

Another aspect of red autumnal leaf colours that may locally or regionally influence their expression is disease (Sinkkonen, 2008). Accordingly, Sinkkonen (2008) showed that in Norway, maple (*Acer platanoides*) reddish leaf colours were more frequent in partially dead trees than in healthy ones. Reddish leaves were evenly distributed in healthy trees, whereas patches of red leaves were typical of partially dead trees. Large patches of fallen red leaves were found beneath or next to dead tree parts. These patches re‐occurred every year. Leaf nitrogen concentration was found to be lower in reddish than in green leaves of Norway maple, but this was similar in both partially dead and healthy trees. Early season reddish colours may thus be used by insects as an indication of low leaf nitrogen and carbon levels but not necessarily as an indication of the whole tree's condition. The influence of tree health should thus also be considered in studies of the evolutionary mechanisms selecting for autumnal leaf colour variation. Interestingly, and in accordance with the case of *Acer platanoides* described by Sinkkonen (2008), leaves in damaged branches in *Populus deltoides* and *P*. *fremontii*, two species with yellow autumn leaves, become yellow before the rest of the foliage (Rood et al., 2000).

## 10 TESTING THE CO‐EVOLUTIONARY HYPOTHESIS

A short and partial review of data directly and indirectly supporting the co‐evolutionary hypothesis was given in Pena‐Novas and Archetti (2021a): ‘The hypothesis has been corroborated by comparative analysis (Archetti, 2009c; Hamilton & Brown, 2001) and empirical studies showing that insects avoid red leaves (Archetti, 2009c; Archetti & Leather, 2005; Döring et al., 2009; Karageorgou & Manetas, 2006), that the amount of red pigment in leaves, and not only in autumn leaves is positively correlated with chemical defences (Cooney et al., 2012; Karageorgou et al., 2008; Menzies et al., 2016), and that insects grow better on plants with green autumn leaves than on plants with red autumn and non‐autumn leaves (Archetti, 2009c; Maskato et al., 2014)’. However, Männistö et al. (2017) tested the co‐evolutionary hypothesis by feeding the larvae of two generalist moth species (*Epirrita autumnata* and *Operophtera brumata*) on the spring leaves of four autumnal red‐leaved woody species (*Populus tremula*, *Sorbus aucuparia*, *Betula pubescens* ssp. *czerepanovii* and *Betula nana*). They found that anthocyanin concentrations in autumn leaves were not correlated with the growth performance of the moths growing on spring leaves in any of the study species, not supporting the co‐evolutionary hypothesis. However, Männistö et al.'s (2017) study was not a direct test of the co‐evolutionary hypothesis because they did not test the choice of landing of egg‐laying females in autumn but rather the growth of caged larvae.

## 11 REVIEWS OF THE FIRST GENERATION OF THE CO‐EVOLUTIONARY HYPOTHESES

Several publications reviewed the first generation of anti‐herbivory hypotheses: Lev‐Yadun and Gould (2007), Archetti et al. (2009a), Archetti et al. (2009a), Schaefer and Ruxton (2011), and Lev‐Yadun (2016). Each review had its strengths and weaknesses and was written with a different focus. Together, they allow a realistic understanding of how it was considered and understood in the past. Schaefer and Ruxton (2011) also provided a delicate and careful review of the experimental voids in many of the hypotheses. Naturally, these earlier reviews lack the later and improved understandings of the subject.

## 12 THE SECOND GENERATION OF HYPOTHESES ABOUT COLOURFUL AUTUMN LEAVES

In the beginning of the year 2008, the arena of the biology of yellow and red autumn leaves was quite a mess, with opposition to several of the physiological and anti‐herbivory hypotheses that tried to explain the phenomenon. Surprisingly, in only about four additional years most of the theoretical conflicts became moderate for some years, although even today we are still very far from even a good understanding of the phenomenon.

The strongest advocate of the defensive (anti‐herbivory) role of red and yellow autumn leaf colouration as a case of Zahavi's handicap principle was Dr. Marco Archetti. After the untimely death of professor Bill Hamilton in the year 2000, Marco Archetti carried the yellow/red autumn leaf co‐evolutionary flag. After publishing several papers about it (Archetti, 2000, 2007a, 2007b; Archetti & Brown, 2004, 2006; Archetti & Leather, 2005; Ramirez et al., 2008), he arranged a small international conference on the autumn leaf colour issue at Oxford's St. John's College in March 2008. At that conference, attended by plant physiologists, plant and animal ecologists, and scientists who specialized in the sensory systems of herbivorous insects, the various and even contrasting views concerning the biology of autumn leaf colouration were presented. Two days of focused discussions vividly illuminated the strong need for a well‐balanced multidisciplinary understanding, and made clear that such a review had to be written and published by the participants. Indeed, a year later it was published in Trends in Ecology and Evolution (Archetti et al., 2009a). Among other things, it became clear to many that yellow and red autumn leaves are in a way two different strategies, although not always and not concerning all anti‐herbivory aspects, and that at least concerning the co‐evolutionary hypothesis that focused on aphids, red and yellow autumn leaves should, from some aspects, be treated separately. Moreover, it also became clear to many that both physiology and anti‐herbivory are involved, that the gains from yellow and red autumn colouration do not stem from only one of these very different function types, and also that not only that aposematic signalling is also involved but also that it largely overlaps with the co‐evolutionary hypothesis, although at that time the level of overlap was not yet well defined. This understanding and the publication of the balanced review were, first, the cornerstone of the second and less naïve generation of hypotheses, and, second, helped in lowering the flames that blazed in the arena of investigating the evolution and functions of colourful autumn leaves. However, this critical understanding and even later progress did not eliminate the need for an even better understanding of the complicated question of the evolution and functions of yellow and red autumn leaf colours. Brown and green autumn leaf colours of deciduous tree, shrub and climber species of the temperate/boreal zones and elsewhere were still not studied much in any case and remained to be studied.

Soon after the publication of Archetti et al. (2009a), Sinkkonen (2009) proposed that UV reflectance should be considered concerning the anti‐herbivory colouration of autumn leaves, because many insects see UV and are repelled by strong UV reflectance. Archetti et al. (2009b) agreed that how leaves look in the UV should be explored, but explained that concerning visual signalling, leaves should be studied as a whole and not as extracted molecules as is done in many studies, because there is a great difference between extracted phytochemicals that reflect UV and whole leaves, and since although certain molecules found in leaves may reflect UV, others may absorb it. Archetti et al. (2009b) showed that measurements of UV leaf reflectance in autumn leaves of more than 2400 species revealed that 99% of them have a maximum UV reflectance of <8.6%, a low value compared with general leaf reflectance and compared with more than 30% UV reflectance in flowers.

After attending the conference, I decided to try to understand why trees with yellow autumn leaves dominate the landscape of northern Europe (e.g. Holopainen & Peltonen, 2002), something that dramatically differs from the dominance of red autumn leaves in landscapes of temperate South and North America and east Asia (e.g. Hoch et al., 2001; Lee et al., 2003). Several years earlier, I did some autumnal fieldwork in eastern USA, and this experience helped me to compare autumn leaf colouration there with that of Scandinavia.

In Finland, the phenomenon of yellow autumn leaves is called ‘Rushka’, and it is very different from what is known from the eastern USA and east Asia. The dominance of yellow autumn leaves in Scandinavia and in other parts of northern Europe is also a very good indication that trees can manage well under low autumn temperatures without the need to turn red. This, along with the many trees and shrubs with yellow autumn leaves in other floras, is good evidence that the physiological role of anthocyanins in withstanding low temperatures is not mandatory and of the possibility that non‐physiological gains may also be involved in red autumn leaf colouration.

**FIGURE 7:**
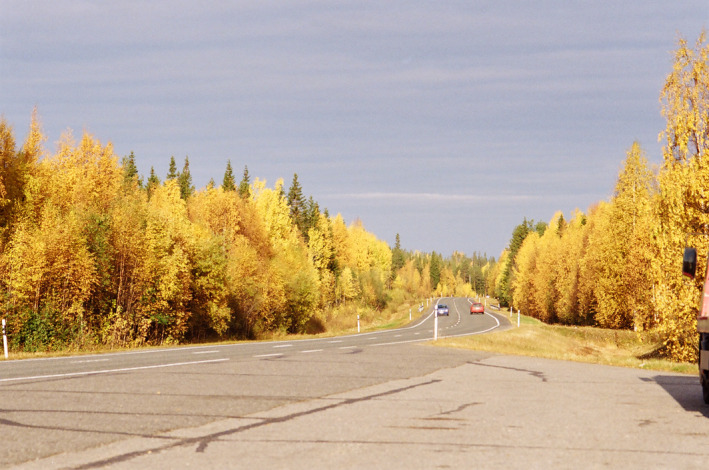
Two golden walls of trees with autumn leaf colouration, central Finland.

**FIGURE 8:**
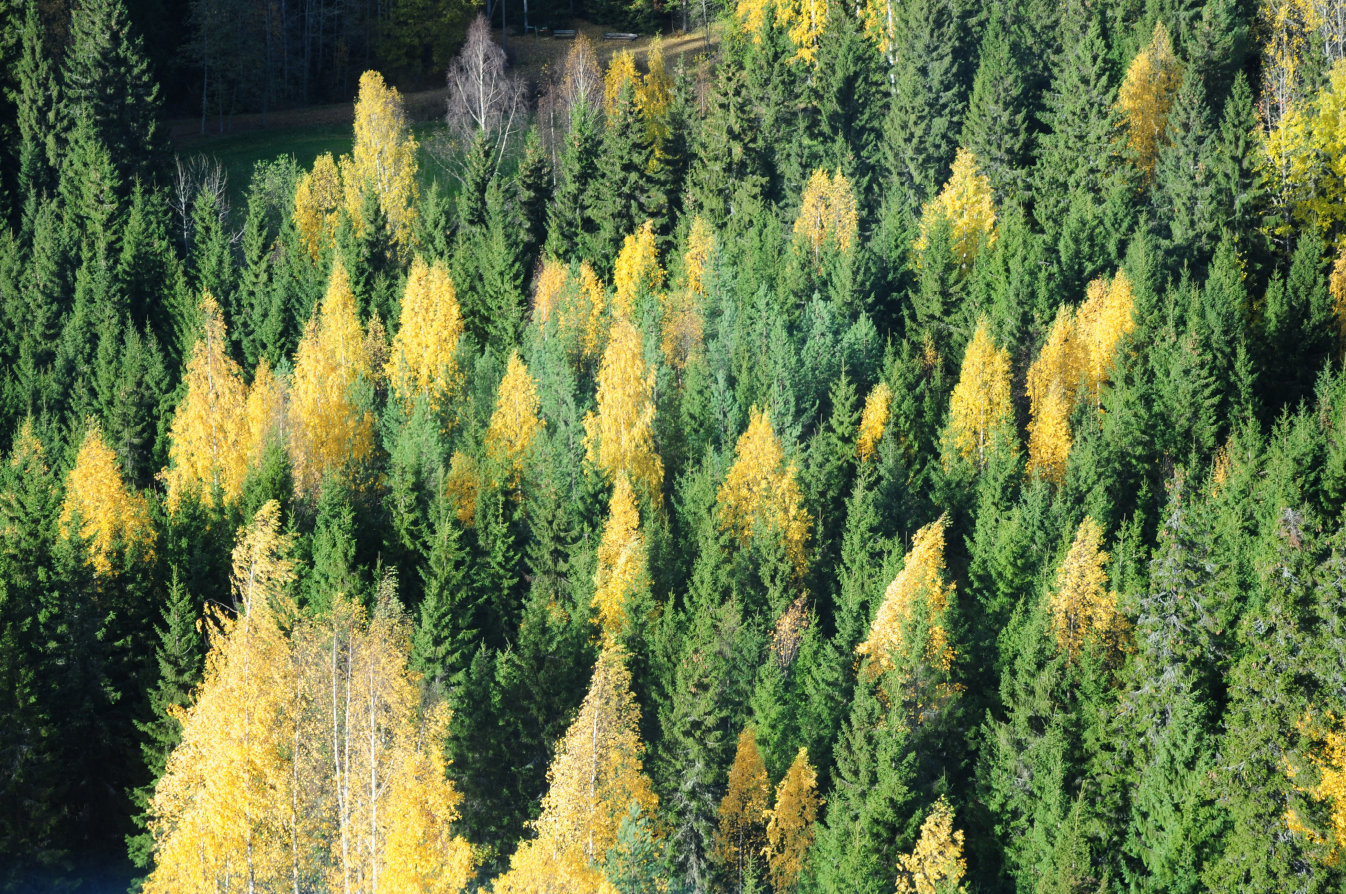
Yellow autumn leaf colouration of *Betula* sp. contrasts with the dark green foliage of evergreen conifers, Kuopio, Finland.

**FIGURE 9:**
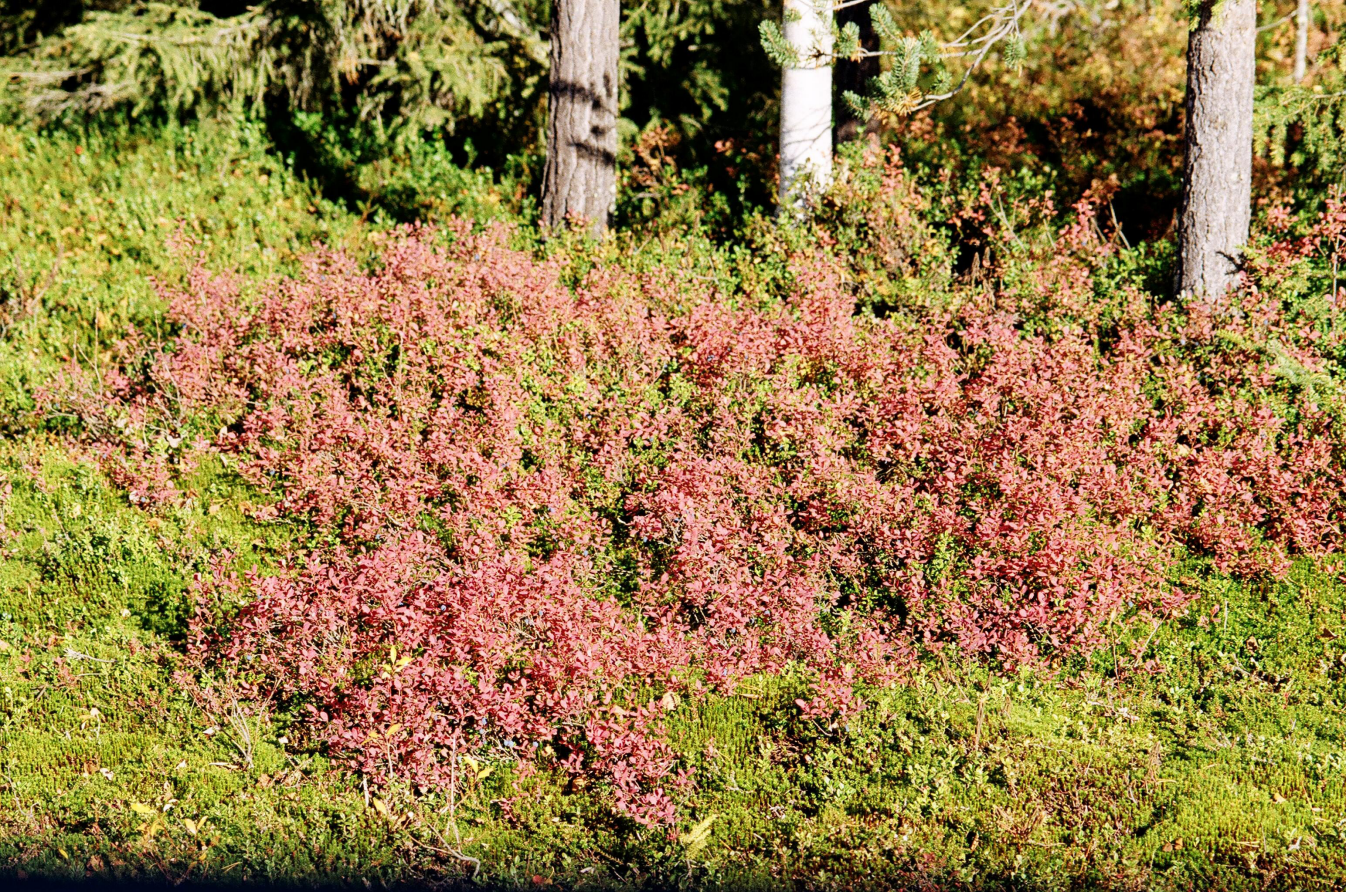
Low shrubs with red autumn leaves found beneath and around trees with yellow autumn leaves or in open areas, not far from the arctic circle in Finland.

**FIGURE 10:**
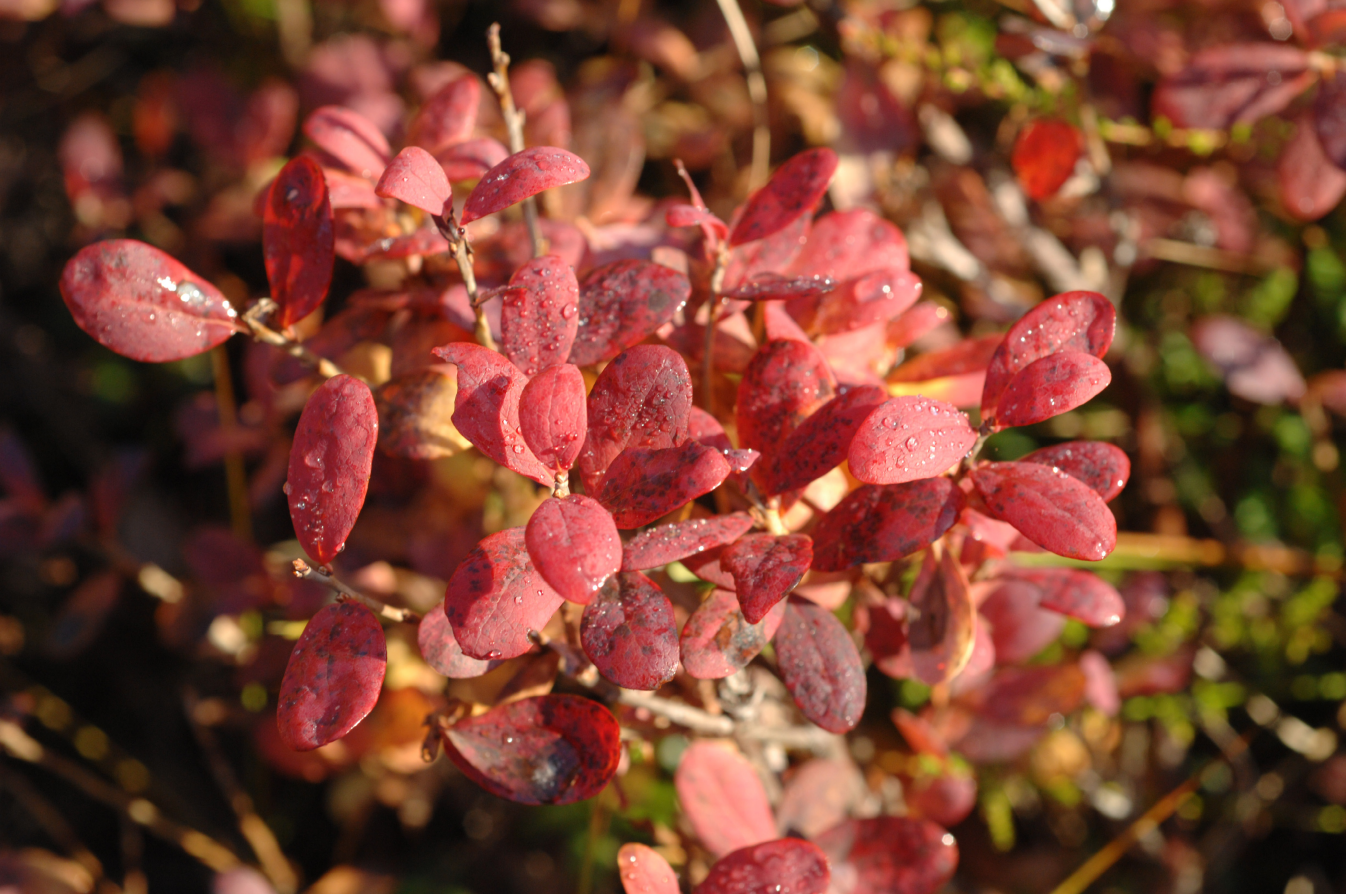
Low shrubs with red autumn leaves in the forest undergrowth, Kuopio, Finland.

The yellow‐gold Rushka belt at its height shifts gradually from the north to the south of Finland in about 2–3 weeks and is visually incredible. Two golden walls of millions of trees with bright yellow‐ or gold‐coloured leaves seen along the roads are shown in Figure 7, or formation of a yellow and green mosaic when the broadleaf trees grow mixed with evergreen conifers is shown in Figure 8. Millions of trees are yellow, but millions of low shrubs of various taxa growing under or next to the yellow trees, under the same climatic conditions, have red autumn leaves (Figures 9 and 10). The contrast between the yellow trees and the red shrubs that grow in exactly the same place was the conspicuous visual key to solving the enigma of the function and origin in geological timescales of yellow *versus* red autumn colouration. Although the trees and shrubs grow in the same location, shrubs and the different specialist herbivorous insects that attack them and select for anti‐herbivory defences, including those associated with red autumn leaf colouration, are exposed to dramatically different conditions during winter than the trees that grow next to them (see below).

**FIGURE 11:**
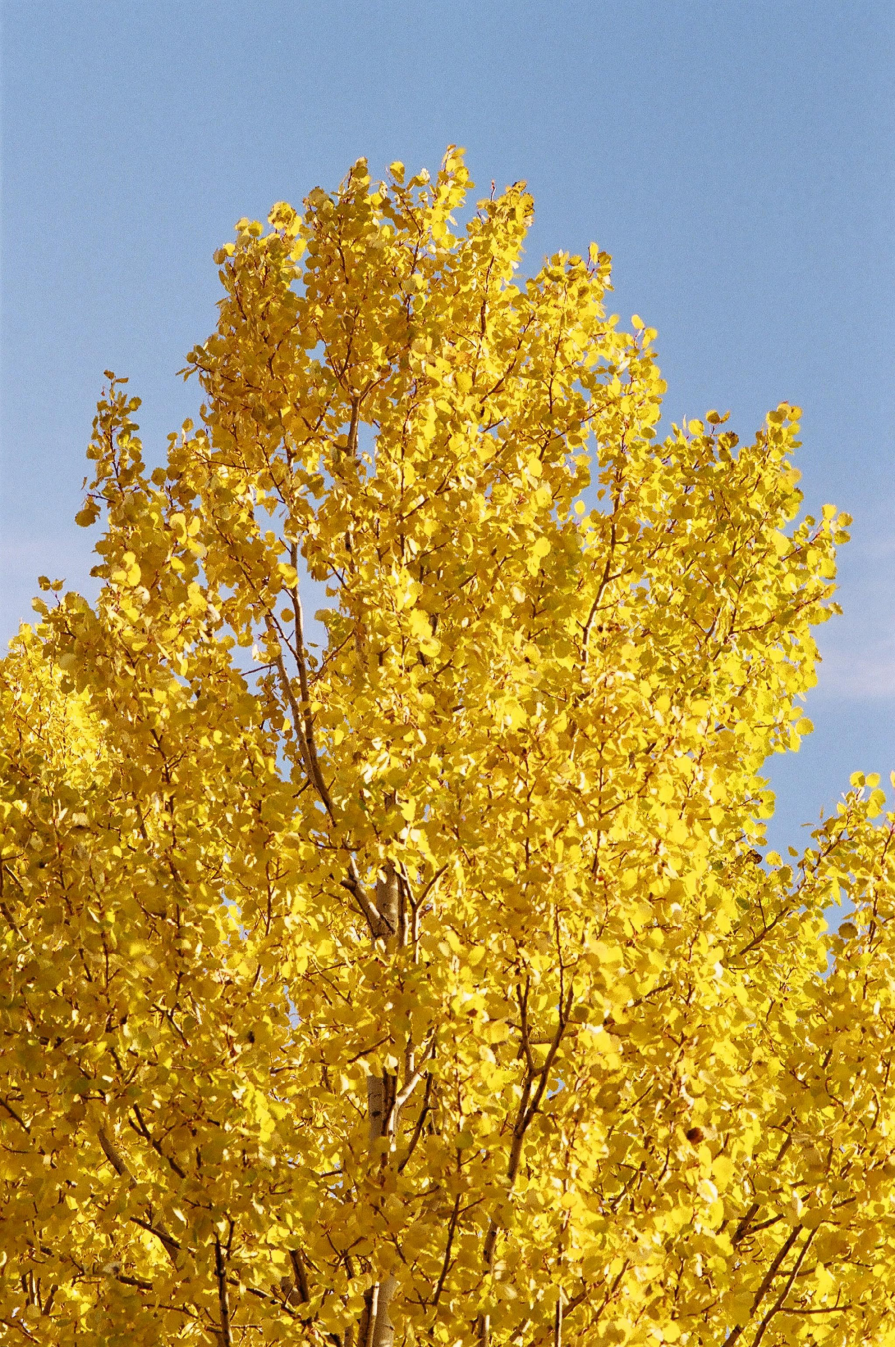
Typical strong yellow/golden autumn leaf colouration of *Betula* sp., Kuopio, Finland.

If adaptations for low autumn temperatures *per se* were the selective agent for red leaf colouration, it would be expected that the Scandinavian autumn tree landscape would have been at least as red as the autumn of eastern North America or east Asia, but it is yellow. Alternatively, yellow autumn leaves would have dominated the autumn landscape of all continents as in Scandinavia, but it is also not so. Therefore, although it is agreed that anthocyanins provide several physiological solutions, especially under low temperatures and simultaneous strong illumination, Lev‐Yadun and Holopainen (2009) suggested that there is clearly no inherited physiological problem in functioning successfully with yellow autumn leaves under the very low Scandinavian autumn temperature, as seen in *Betula* sp. (Figure 11), *Populus* sp. and *Salix* sp., and in the many other deciduous temperate tree taxa that have yellow autumn leaves in Scandinavia and in other parts of the world (e.g. Archetti, 2009a). The possibility that the northern European tree taxa with yellow autumn leaves cannot produce anthocyanins should be dismissed because many temperate taxa with yellow autumn leaves have red colouration in various parts of their canopy, for example during spells of cold weather during leaf flush in spring, or in their reproductive organs. Moreover, many of them regularly have red young leaves in spring and summer (Lev‐Yadun et al., 2012), the very same leaves that turn yellow in autumn!

**FIGURE 12:**
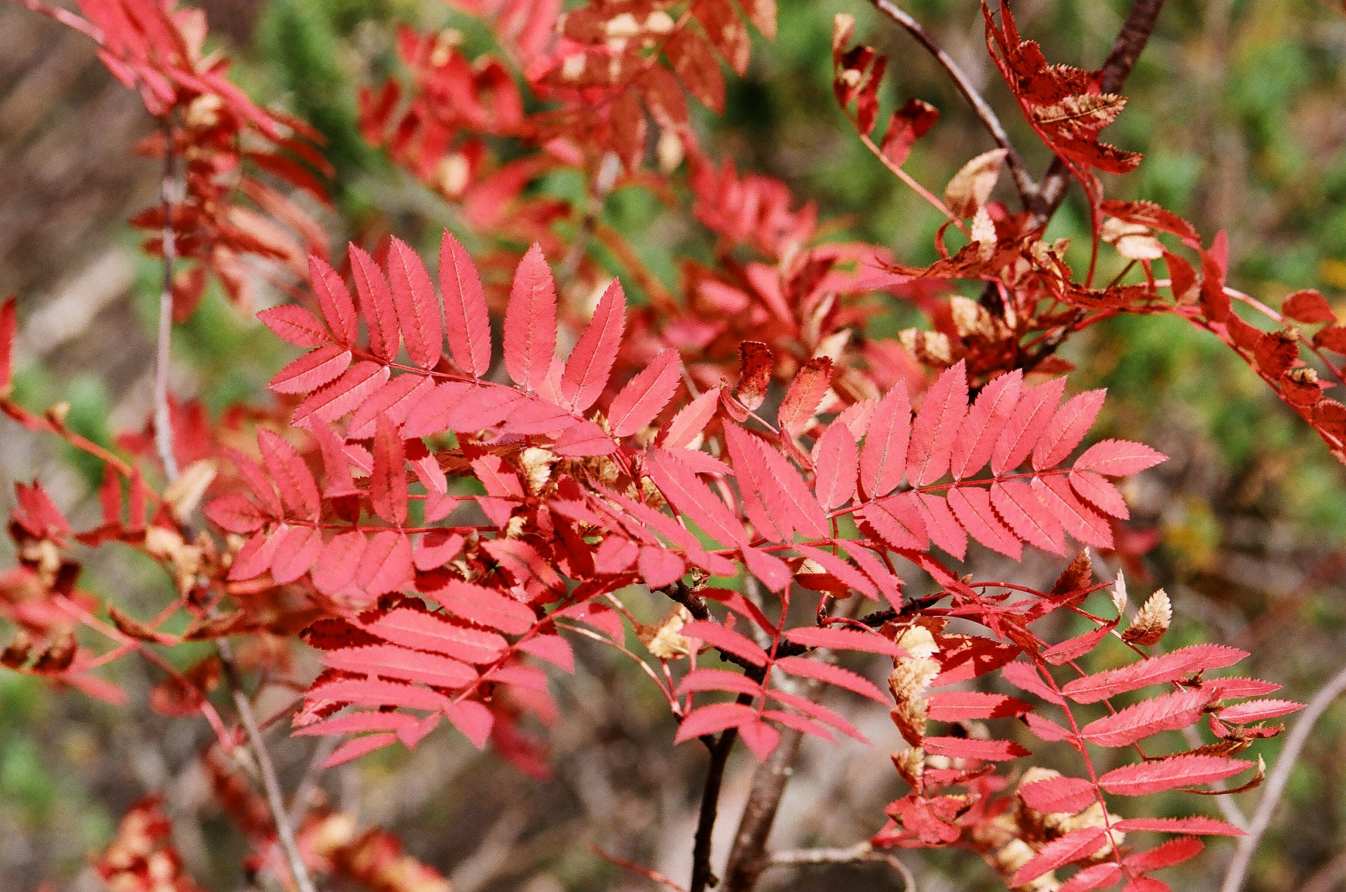
Red autumn leaves of *Sorbus aucuparia*, Kuopio, Finland

Lev‐Yadun and Holopainen (2009) focused on an unexplored aspect of the origin of red autumn leaves in time in relation to past drastic global climatic changes, biological extinctions and migrations starting dozens of millions of years ago, not later than the mid‐Tertiary. They used among other data Archetti's (2009a) published major corpus of 2368 tree and shrub species with red, yellow, brown, and green autumn leaves and found that there are many more species with yellow autumn leaves than with red ones. Archetti's (2009a) findings indicated that the significantly fewer species with red autumn leaves manage on average much better than the species with yellow ones in many non‐European temperate ecologies, and are therefore visually dominant in such landscapes. A broad phylogenetic analysis of the origin of red autumn colouration in those many tree and shrub species indicated that this character evolved independently in temperate woody plants at least 25 times (Archetti, 2009a), supporting again that this is an ancient adaptation. Lev‐Yadun and Holopainen (2009) examined the natural distribution of each of the 290 tree and shrub species with red autumn leaves listed in Archetti et al. (2009a) and found that most of them grow in east Asia and North America. The number of tree species with red autumn leaves in the flora, and even more dramatically in the landscape of northern Europe, is very small. There are only four indigenous tree species with red autumn leaves (*Prunus padus*, *Prunus spinosa*, *Sorbus aucuparia* and *Acer platanoides*) (Figure 12), reaching their northernmost distribution in Northern Europe (Holopainen & Peltonen, 2002), and only 24 such species exist in the whole of Europe. Moreover, *Sorbus aucuparia* has two types, a yellow one and a red one. By contrast, in eastern North America and east Asia both the proportion in the landscape and the actual number of tree species with red autumn leaves are greater. There are at least 89 species in a subset of the woody flora of North America and at least 152 such species in east Asia (e.g. Lee et al., 2003; Lev‐Yadun & Holopainen, 2009).

Lev‐Yadun and Holopainen (2009) used the high prevalence of red autumn colouration of trees in North America and east Asia *versus* the high prevalence of yellow autumn leaves in northern Europe, along with the well‐known gross patterns of migration and extinction during the drastic climatic changes in the Tertiary and especially during the Pleistocene (e.g. Milne & Abbott, 2002) that were briefly described in the beginning of this essay, as a basis for their hypothesis. Lev‐Yadun and Holopainen (2009) proposed that the solution to the problem of the origin of red autumn leaves in general, and their limited distribution in trees of northern Europe in particular, is the well‐known difference in the extinction histories of trees and their herbivores in eastern North America and east Asia (low extinction rate and many Tertiary relicts), and those in Europe (much higher extinction rate). If the origin of red autumn leaf colouration is commonly the result of various ancient Tertiary adaptations of ancient floras to past climates and faunas, which gradually became the current temperate floras (see Axelrod, 1966), then, although physiological adaptations are habitat‐ and climate‐dependent, and a geographical shift when the climate changes is sufficient to allow trees to prosper, the expression of costly defences from herbivory involving the production of red leaves is dependent on a continuous herbivore pressure. However, the current anti‐herbivory adaptations of any flora may reflect not only the current herbivore fauna and their predators and parasites but also many extinct animal species, both vertebrates and insects (see Janzen, 1986). Lev‐Yadun and Holopainen (2009) suggested that not only did most tree species with red autumn leaves become extinct in Europe along with many other species, but when many of their specific herbivores and general herbivores also became extinct, the driving selective agents for a costly defensive red autumn colouration also declined. In any case, the loss of the anti‐aphid function of red autumn leaf colouration was further supported by the findings of Archetti (2009c) for apple trees with red wild‐type *versus* yellow leaves in domesticated ones, and in transgenic apple trees expressing high anthocyanin levels where caterpillars preferred green over red leaves in feeding choice experiments, and grew less when fed with red apple leaves (Markwick et al., 2013).

A strong support for the hypothesis presented by Lev‐Yadun and Holopainen (2009) of an ancient Tertiary origin of red autumn colouration stems from the fact that dwarf shrubs with red autumn leaves, rather than red‐leafed trees, dominate Scandinavia and are also common in other boreal regions, for instance in Alaska. There is a critical difference in the sensitivity of trees and shrubs to extremely cold periods and to extinction during the ice ages, and later to the ability of colonization when warming and re‐colonization of ice‐free territories occurred. The trees and the insects that occupy their bark in winter, usually as eggs (Bale & Hayward, 2010; Dixon, 1977), are exposed to extreme low temperatures that in cold periods may be much lower than −30°C and even as low as −50°C, and therefore, the insect eggs usually die (Niemelä, 1979; Strathdee & Bale, 1998). Many mature boreal trees can withstand much lower temperatures (Strimbeck et al., 2015). By contrast, the low shrubs and their overwintering insects are covered during winter by a snow blanket and a natural igloo, and the shrubs and their insect enemies are thus insulated by the snow from most if not all of the terribly low temperatures. It resulted in the relaxation of the selection for costly chemically defended red autumn leaves on northern European trees. However, the insect‐induced selection for red leaves as defence from herbivory continued for the snow‐insulated low shrubs. In addition, the boreal shrubs can also manage in much less productive habitats than trees, because they manage with shallow soils, or soils that are seasonally or permanently frozen already at a shallow depth. Shrubs and their specialist enemies thus could find more and much larger refugia than trees in periods of glaciation. All these differences allowed shrubs with red autumn leaves and their insect herbivores to escape extinction during periods of glaciation in the Pleistocene, under conditions in which most trees and most of their specialized enemies could not survive, thus continuing their role in selection for red and defended autumn leaves in shrubs.

## 13 THE SHARED AND SEPARATE ROLES OF APOSEMATIC (WARNING) COLOURATION AND THE CO‐EVOLUTIONARY HYPOTHESIS IN DEFENDING AUTUMN LEAVES

Archetti (2000), in the first paper presenting the co‐evolutionary hypothesis, specifically rejected the possibility that these leaves are visually aposematic in his discussion of the defensive signalling by red and yellow autumn leaves towards aphids. In some of the other studies that favoured the co‐evolutionary signalling hypothesis (Archetti, 2007a, 2007b; Archetti & Brown, 2004, 2006; Archetti & Leather, 2005; Brown, 2005; Hagen et al., 2003, 2004; Hamilton & Brown, 2001), aposematism was not discussed. Interestingly, several other scientists (Chittka & Döring, 2007; Gould, 2004; Karageorgou et al., 2008; Karageorgou & Manetas, 2006; Lee, 2002; Lee & Gould, 2002; Manetas, 2006; Schaefer & Rolshausen, 2007a; Sherratt et al., 2005) interpreted the co‐evolutionary hypothesis of autumn colouration presented in the papers by Archetti (2000), Hamilton and Brown (2001), and Archetti and Brown (2004), as a case of warning colouration (actually meaning aposematism, but without using this term), in spite of Archetti's (2000) differing view. The initial negative view about aposematism (e.g. Archetti, 2000) was changed in the updated (second generation) version of the co‐evolutionary hypothesis, which also includes aposematism (Archetti et al., 2009a). Archetti et al. (2009b) and Archetti et al. (2009a) accepted that there is an overlap between the co‐evolutionary and visual aposematic hypotheses, but the fine details and degree of overlap of the two hypotheses were only partly elaborated on at that time, and a more detailed view was given by Lev‐Yadun (2010).

Lev‐Yadun (2010) proposed that the relationships between the original (Archetti, 2000; Archetti & Brown, 2004; Hamilton & Brown, 2001) definition of the co‐evolutionary and the aposematic hypotheses are as follows: The co‐evolutionary hypothesis concerning red and yellow autumn leaves indeed equals aposematism for various herbivorous species for which it is effective because the leaves are toxic or low in nutrients. It is not so for the combination of non‐toxic and nutritious autumn leaves just starting to change from green to yellow towards many aphid species that are visually attracted to yellow leaves (e.g. Holopainen & Peltonen, 2002; Wilkinson et al., 2002). Furthermore, Lev‐Yadun (2010) suggested that the updated second generation of the co‐evolutionary hypothesis (Archetti et al., 2009a) is actually a mixture of two subtypes of aposematism: (1) classic aposematism towards animals that refrain from attacking at the time of signalling, and (2) a type of delayed aposematism, because the purpose of the signalling by colourful autumn leaves is also to deter from egg laying in autumn, and the target insects are not only the mature autumnal egg laying female aphids but also the ones that would hatch from the eggs in the following spring. The production of defensive volatile signals by yellow autumn leaves (Blande et al., 2010; Holopainen, 2008; Holopainen et al., 2010) makes it possible that for at least certain tree species with yellow autumn leaves, the co‐evolutionary hypothesis may operate even towards aphids that are in general visually attracted to yellow leaves, but in such cases, the repellence is done mostly or even only *via* olfactory rather than *via* visual aposematism. A careful examination of both plant and insect species in each studied case is required in order to make a precise classification of the signalling: simple immediate defence by aposematism/co‐evolutionary or a combination of these two strategies (see Holopainen et al., 2009).

## 14 SPRING *VERSUS* AUTUMN OR YOUNG *VERSUS* OLD LEAF COLOURS: EVIDENCE FOR DIFFERENT SELECTIVE AGENTS

Concerning the current leaf herbivore fauna and their potential role in selecting for defensive characters of young spring and old autumn leaf colouration, it is certain that spring and autumn herbivore faunas are in general not identical (e.g. Dixon & Hopkins, 2010; Feeny, 1970). It is well known that in the tropics, young leaves suffer most of their damage from chewing herbivores, although mature and old ones suffer much less chewing (Bixenmann et al., 2013; Coley, 1980; Coley & Barone, 1996; Kursar & Coley, 2003). In autumn leaves, or in old leaves in any season, most of the damage is inflicted by aphids that suck free amino acids and other soluble resources, and leaf tissue consumption by chewing insects is usually much less important (e.g. Caldwell et al., 2016; Coley & Barone, 1996). Because of the differences in types of herbivore damage done to young and old leaves, it is expected to allow disruptive evolution in various leaf characters, including defence by leaf colour.

Lev‐Yadun et al. (2012) examined the possibility that in many woody species different selection agents may have operated on spring *versus* autumn leaf colouration. If the same selective agents (physiological, herbivory or pathological) operated in spring and autumn, it was expected that when young spring leaves are red, they should always be red in autumn, and if young spring leaves are green, they should always be yellow, brown or green in autumn. Alternatively, if defence from herbivory and pathogens rather than only physiology or only developmental constraints also selected for the colouration, spring and autumn colours of the same species may be more random; that is, in many species spring leaf colour should not be directly related to autumn leaf colour and *vice versa*. It is well known that young leaves may suffer much more herbivory damage than mature leaves because of the nutritive superiority of young leaves and because they are mechanically much softer than mature ones (e.g. Coley, 1980, 1983; Coley & Barone, 1996).

Lev‐Yadun et al. (2012) documented the presence of red leaf colour in young and old leaves of a total of 94 woody plant species belonging to three different floras (Finland, central Japan and Israel). This was done in order to allow for a broad ecological and evolutionary spectrum and avoid the risk of documenting convergent local adaptation or phylogenetic relatedness‐based adaptations, risks for a bias that exist when a single flora is studied. Leaves were designated red when red was present even only in parts of the leaves, or not in the whole population. Leaves were not designated red if red was found only next to insect damage (one species in Finland). Lev‐Yadun et al. (2012) found that in many cases spring leaf colour was associated with but not always identical with autumn leaf colours. Green spring leaves were almost exclusively associated with yellow or even with green autumn/senescing leaf colours. Species with red autumn/senescing leaves almost always had red spring leaves. However, about half of the species with red spring leaves had yellow autumn/senescing leaves. Brown autumn/senescing leaves were not common in the species studied.

Lev‐Yadun et al. (2012) found that on average, in about 70% of the studied species, spring and autumn/senescing leaves have different colours, and in about 20% of the studied species autumn/senescing leaves have more than one colour. Unfortunately, it is unknown what the various historical agents selecting for young *versus* senescing leaf colour were for any of the species. The data presented in Lev‐Yadun et al. (2012) strongly supported the hypothesis that in woody dicotyledons, there are many cases where the selective agents on spring *versus* autumn/senescing leaf colouration were different.

Anatomical evidence for different selective agents in young *versus* senescing leaf colouration was available from a broad study of ontogenetic changes of anthocyanin and betacyanin expression in tropical plants (Lee & Collins, 2001). Of 399 angiosperm species, 44.9% produced anthocyanins in young leaves, but only 13.5% during senescence. Of the 35 species that produced anthocyanins in both young and old leaves, several species had different tissue red pigment distribution in spring *versus* autumn leaves (Lee & Collins, 2001). Similarly, in two temperate species, *Corylus avellana* and *Acer platanoides*, the anthocyanins were produced in the epidermis in spring (a better location for signalling), and in the palisade parenchyma (a better location for scavenging ROS) in autumn (Merzlyak et al., 2008).

## 15 HOW DID THE RED AUTUMN LEAF CO‐EVOLUTIONARY HERRING LOSE ITS RED COLOUR?

The co‐evolutionary hypothesis (Archetti, 2000; Archetti & Brown, 2004; Hamilton & Brown, 2001) was awarded two red herrings: (1) Schaefer and Wilkinson (2004), and (2) White (2009). This issue was discussed by Lev‐Yadun and Holopainen (2011) and is given briefly below.

The first red‐herring tag to the co‐evolutionary hypothesis (Schaefer & Wilkinson, 2004) was based on the claim that the red colour may serve as camouflage towards red‐light‐insensitive insects, on the physiological roles of anthocyanins and on the lack of specific data about anti‐herbivory. Actually, the data presented by Hamilton and Brown (2001) about the smaller aphid diversity on tree species with red autumn leaves than in species with yellow ones were sufficient to indicate anti‐herbivory but was not understood then and not always even 20 years later (e.g. Renner & Zohner, 2022). Dr. H. Martin Schaefer was the most critical figure towards the first generation of the autumn leaf co‐evolutionary hypothesis (e.g. Rolshausen & Schaefer, 2007; Schaefer & Gould, 2007; Schaefer & Rolshausen, 2006a, 2006b, 2007a, 2007b; Schaefer & Wilkinson, 2004). His comments, along with comments by others, helped to formulate the improved second generation of the co‐evolutionary hypothesis (i.e. Archetti et al., 2009a). Later, in a very important book about plant‐animal signalling (Schaefer & Ruxton, 2011), the tone about the issue there (page 168) was dramatically more moderate: ‘At the moment, Hamilton's suggestion of co‐evolutionary signalling between plant and herbivore remains a plausible candidate mechanism…’. This welcome change, reflecting a decade of considerable scientific effort by many, and significant progress in understanding this issue, seems to remove the first and major red‐herring tag (e.g. Schaefer & Wilkinson, 2004) from the co‐evolutionary hypothesis.

The second red‐herring tag to the co‐evolutionary hypothesis (White, 2009) was based on a partial misunderstanding of the typical ontogeny of red autumn leaf colouration. White (2009) posited that leaves turn red after they are yellow, and his logic argument was that when the leaves turn red, they have already lost their nitrogen in the form of free amino acids during the yellow stage and that there is no need for defensive signalling at that late red stage of leaf life. White's (2009) statement was partly right and partly wrong, as the situation of changing autumn leaf colour from green to yellow and later to red is certainly not the rule. Red autumn leaves usually turn red from a green state and not from green to yellow and later to red. Leaves in parts of the canopy of various species can indeed change colour from green to yellow because they are shaded, although other leaves in the same canopy are exposed to the sun change colour from green to red. Altogether, there was no reason to tag the co‐evolutionary hypothesis as a red herring on this basis.

## 16 THE THIRD GENERATION OF HYPOTHESES AND THE CURRENT DEBATE

When as a reviewer I read Renner and Zohner's (2019) manuscript, I had the feeling of *déjà vu*. The pre‐Archetti et al. (2009a) debate between plant physiologists and ecologists followed by partial ignorance of anti‐herbivory was presented there again, as if no decade of progress and a much more delicate understanding of that issue had occurred. That paper claimed to present a comparative analysis of several aspects of the physiology of many deciduous species from three continents in order to show that the photoprotective hypothesis is the right and only explanation for the evolution of red autumn leaves. The Renner and Zohner (2019) manuscript had, however, various problems, only some of which were spotted by its reviewers, and even these were not always corrected by the authors as suggested, so some of the obvious problems remained in the published paper, and I will address them below. It also included several miscitations (see below), a problem that continued in their subsequent paper (e.g. Renner & Zohner, 2020). Luckily, the problems in that paper stimulated others to respond. Since Renner and Zohner (2019) did not pay much attention to anti‐herbivory, and the given attention was problematic, it understandably caused Marco Archetti to respond with a letter to the editor (e.g. Pena‐Novas & Archetti, 2020a). This was followed by several additional responses in the New Phytologist and in the Journal of Evolutionary Biology. The responses and the responses to the responses stimulated others to contribute new data and new hypotheses and also to suggest critical corrections to several common previous mistakes (see below), discussions that illuminated both old and new important aspects, especially of the physiology of red and yellow autumn leaves and not much about anti‐herbivory.

Renner and Zohner's (2019) paper compared the existence of anthocyanins and xanthophylls in autumn leaves of deciduous trees from Europe, eastern North America and east Asia with some climatic variables. Renner and Zohner (2019) classified plants that express both yellow and red autumn leaves as a separate group rather than considering them as red ones. It seems to be one of their several basic mistakes, because the ability to produce red autumn leaves is what is important from the genetic, physiological, anti‐herbivory and evolutionary points of view. Moreover, it is well known that many species that have red autumn leaves may also have yellow leaves when the leaves are shaded (e.g. Renner & Zohner, 2019, Figure 1), and having a mixture of yellow and red in the canopy should not alter the classification from red (see below the discussion by Hughes et al., 2022). This mistake alone is sufficient to stop discussing the Renner and Zohner (2019) paper and dismiss it altogether because all their results and conclusions are based on wrong autumn leaf colour classification data, but I will continue. First, they claimed that the geographical distribution of species with yellow and red autumn leaves in eastern North America defies evolutionary (anti‐herbivory) explanations, and this also seems to be a basic mistake. Although they specifically dismissed Lev‐Yadun and Holopainen (2009) without explaining their position point by point, as described above, our paper explained it. There is no reason to think that the current geographical distribution of yellow and red autumn leaves in eastern North America, which reflects four centuries of intensive human activity not less and probably even more than natural climatic‐related processes (see below), defies the several anti‐herbivory explanations mentioned earlier: camouflage, aposematism, undermining herbivorous insect camouflage, signalling that the leaves are going to be shed soon, and certainly the co‐evolutionary hypothesis that overlaps aposematism, several defensive options that are independent of any specific geography. Second, Renner and Zohner (2019) posited by using solar irradiation maps that eastern North America and east Asia receive higher sun irradiation than Europe. Third, they posited that eastern North America experiences higher temperature fluctuations in autumn, resulting in more cold snaps there during leaf senescence. Fourth, by citing their previous studies (e.g. Panchen et al., 2014; Zohner et al., 2017; Zohner & Renner, 2017), they said that in eastern North America the vegetative period (phenology) is 3 weeks shorter, favouring a larger investment in pigments that allow a prolonged period for nutrient resorption before leaf abscission, explaining the higher prevalence of species with colourful autumn leaves in eastern North America than in Europe. Their conclusion was that anthocyanins and xanthophylls in autumn leaves of trees and shrubs from eastern North America mainly serve photoprotection. However, their phenological data emerged from measurements of a small number of individuals per species in botanical gardens, which as we know from previous publications about autumn leaf colour variability (e.g. Silfver et al., 2015; Sinkkonen et al., 2012) cannot represent the full and correct phenological repertoire of any deciduous tree species. Renner and Zohner (2019) based their phenological analysis on the excellent but still quite partial Munich Botanical Garden's woody plant species collection. Considering that impressive but partial species collection as representing the proportion of species with yellow or red autumn leaves in eastern North America and east Asia, their phenological variation, or their growth conditions in their region of origin, was problematic from the outset. They claimed that the Munich Botanical Garden's woody plant collection included 96% of the European winter deciduous species, 49% of the North American ones, and 61% of the Asian ones. However, the number 729 of deciduous temperate tree species used by Renner and Zohner (2019) originated from a paper by Fine and Ree (2006). The origin of this number was in a book chapter (Latham & Ricklefs, 1993) cited by Fine and Ree (2006). Latham and Ricklefs (1993) included in their study only trees and no shrubs and climbers. Thus, the percentage of non‐European woody species out of their floras in the Munich collection is even smaller than the 49% and 61% figure they gave. Moreover, studying many species outside their natural habitat and growth conditions may give not only false phenological results, but also because some species change their autumn leaf colours under non‐natural conditions (e.g. Hughes et al., 2022), the basic data about it in Renner and Zohner (2019) are incorrect. On top of this, both latitude and altitude greatly influence the length of the vegetative period, and this was not taken into consideration by Renner and Zohner (2019), who pooled results for whole continents into a single average.

Another problematic issue in the Renner and Zohner (2019) paper is the climatic data presented in their figure 5a. They presented a map of large parts of the world, showing the average short‐wave irradiation for September of the years 1901 to 2010, citing Beer et al. (2014) as the source of the data. However, Beer et al. (2014) deals only with Europe, and the origin of data outside Europe is unclear. Moreover, the Beer et al. (2014) paper used several critically problematic assumptions about the ability to reconstruct solar irradiation rather than using only actual measurements, which cast doubt on the accuracy of their data. In addition, several aspects of the Beer et al. (2014) paper were based on Weedon et al. (2011), which also had several layers of assumptions for huge parts of the world and during many decades for which there are no actual meteorological measurements between the year 1901 and the year 1958. Simply, detailed measurements of short‐wave irradiation over much of the earth were impossible for more than half of these 110 years. Thinking for instance that there are enough reliable measurements from Siberia and from other parts of central, east, and northern Asia before World War I, and until modern satellites provided information *via* remote sensing, when most of that huge region was inaccessible and not monitored at ground level, seems to be a clear mistake. Moreover, in days when thick cloud blankets cover huge areas, as happens during autumn, they protect the forests from strong sun irradiation. Altogether, there is no way to consider all types of meteorological data used by Renner and Zohner (2019) as reflecting reality, and all their calculations, statistics, physiological and evolutionary conclusions that are based on this reflect nothing but naïve innocent reliance on non‐data. In addition, and of great and even critical importance for many species and habitats, sun irradiation differs a lot in habitats of southern (facing south) aspects *versus* those of northern (facing north) aspects, especially during autumn when the sun in temperate and boreal latitudes is quite low in the horizon. An excellent demonstration of the importance of aspect concerning sun irradiation for woody plants can be seen in Switzerland and southern Germany, where *Vitis vinifera* orchards are planted on southern sides of many hills in order to expose them to more sun irradiation and improve grape production, chemistry and consequently wine quality. Thus, even if the ‘big data’ about short‐wave sun irradiation over continents was real, as considered by Renner and Zohner (2019), there is a great problem with what they suggest. Even in areas adjacent to each other in hill or mountain regions, there is a strong difference in sun irradiation because of the aspect. Daubenmire (1974, page 217) in his classic book explained: ‘The direction and slope of the land surface caused marked variations in the intensity and daily duration of insolation. In general the temperature aspect of this topographic factor is probably more important than the light aspect. However, on steep poleward slopes direct sunlight may be completely lacking at noon so that plants must rely heavily on sky light, which is only about 17% as intense as the light received by a surface level enough to get full direct lighting’. In Israel for instance, this is a very important factor determining species distribution beginning in desert habitats, which receive about 100 mm of average annual rainfall, to the much more humid Mediterranean district inhabited by several autumn/winter deciduous tree and shrub species under an average annual rainfall of 500–800 mm. The aspect influences the local distribution of more thermophilus and less thermophilus species (Nevo, 2012; Zohary, 1962), an adaptation that in many cases goes along with different sensitivity to water supply. Ignoring aspect's influence may lead to irrelevant and erroneous results. In addition, treating huge areas stretching over thousands of kilometres, such as a continent, as a single unit, lowers the resolution that can be achieved by a more careful sampling or by compiling of published data and their analysis. The gross subdivision of the vegetation and floras of eastern North America, Europe and east Asia into several very different biomes has been known for many decades (Breckle, 2002), and grouping Mediterranean Europe, or even western or central Europe with Scandinavia is also a significant mistake. The same mistake applies to treating the forests in the northern and southern parts of eastern North America and in those in east Asia as single units. Moreover, if there are differences in leaf colour distribution between northern and southern aspects, paying attention to it may not only give much more reliable results, but also help in understanding the physiological differences between trees with yellow *versus* red autumn leaves.

A different theoretical problem with the Renner and Zohner (2019) paper is related to the current geography of deciduous trees in eastern North America, Europe and east Asia. The virgin forest of eastern North America, which had about 12 millennia to arrive at a type of non‐perfect equilibrium (because for instance the different dispersal abilities of taxa with heavy seeds such as oaks *versus* wind‐borne winged seeds of conifers and dicots; Davis et al., 1986) with the post‐glacial conditions in the huge areas freed from thick ice cover and opened for colonization in the early Holocene. These virgin forests were almost totally wiped off by human activity since the 17^th^ century (Williams, 2003). The original composition of the forest, as well as the data about the distribution of the various species in relation to local environmental factors, was mostly lost when the original native forests were cleared. The current forests there represent founder effects and processes of succession from an agroserra (abandoned farmland) and from other man‐made disturbances (e.g. Fain et al., 1994; Hibbs, 1983; Horn, 1971; Williams, 2003). Therefore, measuring tree characters and several environmental variables, comparing them and trying to identify trends as if the current forests of eastern North America are well‐adapted native forests that represent an equilibrium with the climate, are prone to give a false picture. Man‐made disturbances and succession of pioneer and late‐successional species may be much more important than the current or past local climates. Virgin forests also do not exist in southern, western, central and most of northern Europe because of millennia of human activities (e.g. Williams, 2003). When also ignoring the combination of critical factors such as aspect, recent and current post‐disturbance succession, and treating whole continents as one unit in spite of enormous intra‐continent climatic gradients and differences, the continent‐level suggestions by Renner and Zohner (2019, 2020) (and also in some of the responses to their papers) as representing only adaptations towards natural meteorological/climatic conditions should be considered with great caution.

In spite of the above critical comments, altogether, the wave of responses and the new facts and hypotheses that were published because of the stimulation by the Renner and Zohner (2019) paper, contributed to the understanding of the biology of autumn leaf colouration more than their original paper, a repeat of what happened after the publication of the co‐evolutionary hypothesis by the Hamilton group (Archetti, 2000; Archetti & Brown, 2004; Hamilton & Brown, 2001). Interestingly, the papers by Renner and Zohner (2019, 2020) and some of the responses were not focused directly only on supporting the photoprotection or the co‐evolutionary hypotheses but rather on showing that the advocates of the other hypotheses are wrong. This led some of the authors to conduct research or analyses in scientific territories in which they are not at their best. Luckily, it caused experts of the other scientific territories to respond and to clarify important things.

The first response to Renner and Zohner's (2019) review was by Pena‐Novas and Archetti (2020a), which presented the gross biogeography of autumn leaf colouration in woody plants of eastern North America and Europe. Although the number of species in their sample was larger than that of Renner and Zohner's (2019), it also included many evergreens rather than focusing on deciduous woody species. Therefore, I do not cite the numbers given there. Pena‐Novas and Archetti (2020a) proceeded with an explanation why comparing floras does not reveal adaptive mechanisms. This statement is not always correct, because convergent evolution in several continents can illuminate adaptations and their selective agents, for example sclerophyllous leaves in Mediterranean woody evergreens in western Asia, southern Europe, North and South America, North and South Africa, and west Australia (Kummerow, 1973). Pena‐Novas and Archetti (2020a) also mentioned several additional temperate regions with a high frequency of colourful autumn leaves (Patagonia, central Asia, the Russian far east), and several temperate regions with low frequency of colourful autumn leaves (western North America, Australia, New Zealand), and argued that woody plants in eastern and western North America may need the same photoprotection (because of being in the same latitudes and under the same level of sun irradiation). Pena‐Novas and Archetti (2020a) said correctly that comparing species with red leaves with species with non‐red leaves is better than averaging whole floras. They even proposed that studying intraspecific variation may be even more illuminating, as was done for wild and domesticated apple (Archetti, 2009c), and for several other tree species (see Baisden et al., 2018). Pena‐Novas and Archetti (2020a) gave a short overview of papers both supporting and contra the photoprotective function. This short review looked in a first glance as a balanced short presentation of the conflicting results concerning photoprotection by anthocyanins, but it was addressed without going deep into the physiology. Later, Agati et al. (2021) and Hughes et al. (2022) pointed to several critical mistakes in the earlier references discussing photoprotection that were cited by Pena‐Novas and Archetti (2020a), and therefore to problems in some of their conclusions. Naturally, Pena‐Novas and Archetti (2020a) presented (shortly) the co‐evolutionary hypothesis and cited some studies that support it. Of great importance is the fact that Pena‐Novas and Archetti (2020a) summarized that both photoprotection and the co‐evolutionary hypothesis are the best candidates explaining the evolution of the phenomenon of colourful autumn leaves, but ignored other anti‐herbivory hypotheses that I discussed above.

Back‐to‐back in the same New Phytologist issue, Renner and Zohner (2020) responded to Pena‐Novas and Archetti (2020a). Unfortunately, Renner and Zohner's (2020) short response included several miscitations. Already in the first five lines, they cited Lev‐Yadun and Holopainen (2009) as if we said that eastern North America, with 89 species expressing red autumn leaves, has more such species than east Asia, for which Lev‐Yadun and Holopainen (2009) found at least 152 such species, a mistake that can mislead others. Several lines later, Renner and Zohner (2020) wrote that Pena‐Novas and Archetti (2020a) posited that autumn leaf colours evolved only as a warning signal towards herbivores. This was again a miscitation, since as mentioned only several lines above, Pena‐Novas and Archetti (2020a) concluded that both photoprotection and the co‐evolutionary hypothesis are the best candidates for explaining the evolution of colourful autumn leaves. Renner and Zohner (2020) posited that in the previous 18 years the predictions of the co‐evolutionary hypothesis were never supported. Again, this was a mistake, because trees and other plant types commonly use red leaf colouration as defence from herbivory, and as cited earlier, differences in yellow leaf colour changes were also found to influence insect behaviour, two issues that were reviewed above and ignored by Renner and Zohner (2020). Renner and Zohner (2020) referred again to the possible influence of higher temperature fluctuations in eastern North America compared to Europe and east Asia as they had shown, but again did not take into account the great influence of the aspect not only on temperature but also on the amount of sun irradiation in all wavelengths. Renner and Zohner (2020) combined their data with the Pena‐Novas and Archetti (2020a) data and analysed it in a hierarchical Bayesian framework. Their new analysis allowed them to quantify continental‐scale differences. However, in addition to the severe problems with their climatic and phenological data that were discussed above, actual ecology and evolution of most tree species occur at individual, niche, habitat and mating population levels, and not at continental ones. Renner and Zohner (2020) concluded that their data indicated that there is a need for an ultimate explanation for the lower frequency of red‐coloured autumn leaves in Europe (already disagreeing in their 2019 review with Lev‐Yadun & Holopainen, 2009, who suggested an ultimate explanation that was ignored again by Renner & Zohner in 2020) and also posited that this is also where the co‐evolutionary hypothesis falls short. It is obvious why Pena‐Novas and Archetti quickly responded again.

At that stage, the debate spread from the New Phytologist to the Journal of Evolutionary Biology (Pena‐Novas & Archetti, 2020b), a response that focused on the photoprotective hypothesis. Like Lev‐Yadun and Holopainen (2009), Pena‐Novas and Archetti (2020b) considered in their analysis species that express a mixture of red and yellow autumn leaves as red ones (see also Hughes et al., 2022). Pena‐Novas and Archetti (2020b) indirectly tested the predictions of the photoprotective hypothesis by comparing the climatic parameters in the current geographical distribution of 237 North American tree species (also without considering aspect influences). They also studied only the species included in a recent phylogeny (e.g. Ma et al., 2016), for which climatic parameters were available. Although they found that trees with yellow autumn leaves grow under lower minimum temperatures than species that shed green leaves, there was no significant difference between trees with red autumn leaves and those with yellow ones. Although Pena‐Novas and Archetti (2020b) used a much more accurate species‐specific characters and species‐specific climatic data than Renner and Zohner (2019, 2020), like Renner and Zohner, their sample was taxonomically too small, something that makes it difficult to evaluate their results. The most important outcome of the Pena‐Novas and Archetti (2020b) study, which was not their intended focus, was that they showed the evolutionary flexibility of switching of autumn leaf colour multiple times from green to yellow or red, from yellow or red to green, from red to yellow, and from yellow to red, adding to the data on autumn leaf colour origin given in Archetti et al. (2009a). Such switching shows indirectly that the functional differences between red and yellow autumn leaves may not always be dramatic. The fact that trees with red autumn leaves evolved to become yellow ones and *vice versa* (Pena‐Novas & Archetti, 2020b) may emerge in the near future as a critical opportunity for understanding the genetic and cellular level details of the evolution of autumn leaf colouration. Such cases also have a good potential for a better understanding of the environmental factors involved in the evolution of yellow and red autumn leaf colouration. Moreover, like the multiomics study conducted on *Pistacia chinensis* that revealed the 31 genes of anthocyanin biosynthesis and other genes involved in red autumn leaf colouration in that species (Song et al., 2021), studying the leaf colour switches in species of the same genus, or better, in different genotypes of the same species can illuminate the specific genes and functions involved in such colour switches, that is the specific modes of evolution. In addition, Pena‐Novas and Archetti (2020b) found that species with red autumn leaves grow in habitats that receive significantly higher amounts of precipitation than species with green autumn leaves. They concluded, while not considering some old evidence for potential warming by red colour, that although resorbing degraded chlorophyll in autumn and the consequent unmasking of yellow carotenoids may be an adaptation to low temperatures, the production of red anthocyanins is not.

Concerning the plant distribution issue, both Renner and Zohner (2019, 2020), and Pena‐Novas and Archetti (2020a, 2020b) have similar problems. Neither research groups considered the influence of northern and southern aspects, that is the actual temperature, level of sun irradiation, and humidity, in the specific habitats of many species. A good strategy for testing the role of temperature, sun irradiation, precipitation and evaporation is to compare the species that occupy the northern aspect of a mountain or hill ridge with those of the southern aspect. It can and should be done at the species, genus and local flora levels. Pena‐Novas and Archetti (2020b) assumed that the ranges of the species they studied had not changed over evolutionary time. This was a mistake on two grounds: (1) the frequent huge changes in temperate plant distribution during the Pleistocene following the four enormous waves of glaciation and the warm phases that followed and (2) the processes of forest clearing in eastern North America starting with the European colonization beginning 400 years ago (Williams, 2003), and the forest succession that followed, especially after many farmlands were abandoned in the 20^th^ century.

The fact that Pena‐Novas and Archetti (2020b) concluded that red does not have a function in improving resorbing chlorophyll in autumn leaves was very problematic and surprising in the light of previous although insufficient data to the contrary (see below). It should be remembered that it is very difficult to measure the various roles of anthocyanins in resorption of nitrogen and other resources when some other molecules simultaneously do the same.

In their third contribution to the debate, Pena‐Novas and Archetti (2021a) continued their effort from their previous (Pena‐Novas & Archetti, 2020b) paper to convince that the photoprotection hypothesis is not supported. They examined the nitrogen content of mature *versus* senescent autumn leaves of various colours belonging to 55 tree species (29 yellow, 17 red and nine green). They found no correlation between the presence of anthocyanins and the efficiency of nitrogen resorption. Moreover, they found that nitrogen resorption was more efficient in species with yellow autumn leaves. Again, they concluded that the photoprotective hypothesis (via anthocyanins) is not supported. Their findings should be viewed while remembering that there are many cases in which leaves turn red because of low nitrogen (e.g. Hughes et al., 2022), and that in such cases, there is less to resorb from the outset. Three changes in the experimental design could have make it more convincing. The first and obvious would be to significantly increase the number of species studied, because as the authors themselves say in the discussion, it was too small. The second and also obvious is that although it is impossible to ignore the great importance of nitrogen for species that do not have symbiotic bacteria that fix nitrogen, other resources such as phosphorus, manganese and ions of other elements, and sometimes even carbohydrates, may also be of great importance, but their resorption was not measured. The third potential way to improve that line of study is to compare nitrogen (and other resources) resorption in yellow and red leaves of the same individual tree in trees that express such colour variability. However, this potential way to test the indirect role of anthocyanins in nitrogen resorption may not always work (see Feild et al., 2001).

The most important paper since the year 2000 about photoprotection by red leaf colouration, and not only concerning autumnal ones, was that of Agati et al. (2021), which was the sixth paper in the current debate. First, that paper described the central problem in previously published data concerning the range of light wavelengths absorbed by anthocyanins, a fundamental aspect ignored or misunderstood by many. It lists and explains the problems in the experimental design and in the interpretation in a number of previous papers. When dealing with photoprotection by anthocyanins, the facts detailed and explained by Agati et al. (2021) should have been the starting point of the discussions about photoprotection several decades ago, but unfortunately, it was not understood or practised by many. Agati et al. (2021) gave a detailed description of the absorbance spectrum of anthocyanins, showing that contrary to common belief, they absorb all the way from red to UV. Agati et al. (2021) explained that indeed, anthocyanins have their maximal absorption in the green, but that Pena‐Novas and Archetti's paper (2020a), which followed earlier papers by various plant physiologists, considered green as the only colour that anthocyanins absorb, which is both wrong and misleading. Agati et al. (2021) explained that measurements of the spectral features of anthocyanins, which are usually done under acidic extract solutions (pH 1.5–2.0), differ from vacuolar pH, which is around 5.0, and that this difference shifts their actual absorbance to longer wavelengths. This is the type of irrelevant context problem in many physiological studies that I mentioned earlier in this essay. Agati et al. (2021) strongly criticized Pena‐Novas and Archetti's (2020a) conclusions that there is not enough evidence in support of the photoprotective effects of anthocyanins, saying that Pena‐Novas and Archetti (2020a) were counterfactual. Agati et al. (2021) listed a number of recent papers (years 2014–2021) that showed the photoprotective effects of anthocyanins. Agati et al. (2021) further explained that although Renner and Zohner (2019, 2020), and Pena‐Novas and Archetti (2020a, 2020b) conducted observational field studies for revealing photoprotection, while measuring the light absorbance, correct measurements of actual photoprotection are also needed. Agati et al. (2021) continued with a very detailed explanation of the mistakes in measurements (other than absorbance spectrum) in various studies that were aimed at understanding photoprotection. They stressed that measuring photosynthesis and by this estimating the photoprotective role of anthocyanins only once a day, as was done in most studies, may lead to wrong and misleading conclusions. In their conclusion, Agati et al. (2021) warned against scaling up from cellular/organ levels to whole plants in the forest that interact with multiple environmental stimuli or, in my words, beware the out‐of‐context problem. Agati et al. (2021) explained that Renner and Zohner (2019, 2020) supported their photoprotection hypothesis by using the mechanistic insights given by Gould et al. (2018), who used data from ‘wild‐type’ and anthocyanin‐rich *Arabidopsis thaliana* mutants exposed to excessive light energy. It can be useful to understand and remember that current laboratory *A*. *thaliana* stocks passed many generations of selection under artificial growth conditions and are not ‘wild‐type’ any more (see Lev‐Yadun & Berleth, 2009). Agati et al. (2021) explained that Pena‐Novas and Archetti (2020a, 2020b) challenged Renner and Zohner (2019, 2020) not only by concerns about their understanding of the environmental drivers for anthocyanin biosynthesis, but also on the wrong assumptions by Pena‐Novas and Archetti (2020b) about the light‐absorbing properties of anthocyanins, as reported by Manetas (2006). Agati et al. (2021) suggested that the driving force for the evolution of autumn leaf colours (they should have said red) was to provide cold‐sensitive individuals exposed simultaneously to high sun irradiation and low temperatures with a flavonoid primarily devoted to mitigating photooxidative stress. They said that the accumulation of anthocyanins in peripheral tissues may both protect leaves from excess light and serve as a visual warning signal. Agati et al. (2021) speculated that autumn leaves expressing anthocyanins first evolved in order to defend from various aspects of photoinhibition and only later as defence from herbivory. I tend to partly agree with this suggestion. I do not fully agree because there are red roots and underground shoots that express anthocyanins or betacyanins without being exposed to light.

Pena‐Novas and Archetti's (2021b) response to Agati et al. (2021) focused on what they considered the missing evidence for the photoprotection hypothesis. They agreed with Agati et al. (2021) that there is evidence favouring photoprotection by anthocyanins (as they did in their first contribution to the debate, e.g. Pena‐Novas & Archetti, 2020a). Pena‐Novas and Archetti (2021b) insisted that the issue is not whether there is photoprotection by anthocyanins, but that this is not a proof that photoprotection by anthocyanins is the explanation for the evolution of red autumn leaves. Pena‐Novas and Archetti (2021b) explained that the issue is not photoprotection *per se*, but rather, first the question of nitrogen resorption, and second the interspecific variation. Although these two points are indeed critical, I wish to add that intraspecific variation is also very important in the evolution of any character. Pena‐Novas and Archetti (2021b) posited correctly that the known data about better nitrogen resorption in red leaves are insufficient, and referred again to the contradictory results in various studies. Moreover, they pointed to the view given in Manetas (2006), and in Duan et al. (2014), that anthocyanins are not located in the cell in an optimal place for reducing light levels. This point, which was only partly discussed here earlier, will be further discussed below following the contribution of Hughes et al. (2022) on this specific critical issue. Pena‐Novas and Archetti (2021b) continued by discussing Agati et al.'s (2021) suggestion that low temperatures and strong sun irradiation triggers anthocyanin production in sensitive individuals, which in turn allows better nitrogen resorption. Pena‐Novas and Archetti (2021b) rightly said that concerning this point both they and Agati et al. (2021) said essentially the same but in different words. Pena‐Novas and Archetti (2021b) explained that photoprotection alone does not influence tree evolution because there is no need to protect leaves that are going to be shed anyway unless it bears on tree's fitness *via* more efficient resorption. Pena‐Novas and Archetti (2021b) explained that similar to photoprotection and better resorption in red leaves that prove nothing *per se*, the fact that insect pests avoid red leaves also does not prove the co‐evolutionary hypothesis. Pena‐Novas and Archetti (2021b) posited that Renner and Zohner's (2019, 2020) suggestion that a higher prevalence, that is the percentage in the flora of tree species with conspicuous autumn colours (yellow and red) in eastern North America than in Europe, is not significant (because in Europe, the extinction during the ice ages was the greatest as I explained earlier), and suggested that a more appropriate approach would be to compare species with red and species with non‐red autumn leaves and test if the red ones grow under lower temperatures or under higher sun irradiation. I posit that the percentage or the number of species with red autumn leaves in a flora is probably not the most important factor because this value may just reflect the different levels of the well‐known extinction during the Pleistocene. What is more critical is their importance in the landscape, which is much larger in eastern North America and in east Asia than in Europe. Pena‐Novas and Archetti's (2021b) explained that this is what they examined in their 2020b paper and that they found no difference in growing temperatures between species with red autumn leaves and trees with green or yellow ones, and that these results do not corroborate the photoprotection hypothesis. Since both Renner and Zohner (2019, 2020) and Pena‐Novas and Archetti (2020b, 2021a, 2021b) did not consider the influence of aspect (north *versus* south) on light level, temperature, and water economy, as well as the influences of the recent forest succession induced by human activity in the forests of eastern North America, or even the 12 000 years of the last post‐glacial re‐colonization and the even larger and longer impact of human activity on European forests, their results may not fully reflect the evolution of these species concerning autumn leaf colouration, but rather various natural and man‐made disturbances and many founder effects.

Hughes et al. (2022) was aimed to be the ‘peace‐maker’ and bridge the sharp disagreements between Renner and Zohner that posited that the co‐evolutionary hypothesis is invalid while trying to show that the photoprotective hypothesis is the only explanation for the evolution of red autumn leaves, and Pena‐Novas and Archetti who defended the co‐evolutionary hypothesis while pointing to problems in proving the photoprotective hypothesis but not dismissing it. Hughes et al. (2022) argued that both hypotheses could be equally valid, and even complementary, if one considered soil mineral deficiency in addition to temperature and sunlight as a selective pressure during leaf senescence. Indeed, nutrient deficiencies both increase the need for photoprotection as a result of reduced photosynthetic capacity (Evans & Seemann, 1989; Hikosaka, 2004; Martin et al., 2002) and render the plant a lower quality food source for herbivores (Ball et al., 2000; reviewed in Awmack & Leather, 2002). The most original aspect of Hughes et al. (2022) was dealing with the issue of soil fertility as a factor that influences autumn leaf colouration. They were greatly influenced by the works by Manetas' research group on the connection between N‐deficiency and anthocyanins (e.g. Kytridis et al., 2008). Manetas' research group argued that when red individuals grow among green individuals, they are probably N‐stressed. This hypothesis was tested as the explanation of the simultaneous occurrence of red‐ and green‐leafed Japanese honeysuckle. Indeed, at all three field sites, the red individuals had significantly lower N than the green ones during winter (they turned red only in winter) (Carpenter et al., 2014). Hughes et al. (2022) reviewed a number of previous studies that demonstrated that red‐leafed individual trees tend to exhibit lower % leaf nitrogen than green‐leafed conspecifics, because leaf reddening is a common symptom of nitrogen deficiency in many plant types including herbaceous, woody evergreens and deciduous ones. Hughes et al. (2022) supported this idea by citing multiple studies reporting a connection between the low‐N and high anthocyanins, including Schaberg et al. (2003), who documented more intense and earlier autumn leaf reddening in low‐N individuals of sugar maple, *Acer saccharum*, during leaf senescence. Although it was known and mentioned in some earlier publications that species that have nitrogen‐fixing bacteria, such as *Alnus glutinosa*, shed green leaves, the broad view about soil fertility and autumn leaf colouration was not considered until the Hughes et al. (2022) paper. Hughes et al. (2022) also said that if leaf reddening has indeed evolved in response to low leaf nutrients, and low nutrient trees exhibit greater nutrient resorption than high nutrient trees, then red‐leafed‐deciduous species might exhibit more efficient nutrient resorption than yellow species, but not directly because of high anthocyanin levels *per se*. Hughes et al. (2022) noted that unfortunately none of the studies comparing nutrient resorption efficiencies of red‐ and yellow‐leaved deciduous species properly controlled for leaf mass loss during senescence. However, I suspect that since many autumn leaves emit volatiles (e.g. Blande et al., 2010; Holopainen, 2008; Keskitalo et al., 2005), there is probably no realistic way to control for leaf mass. Hughes et al. (2022) said correctly that a larger study comparing the timing of chlorophyll breakdown and concerted photoprotective strategies for red *versus* yellow ones is needed. Hughes et al. (2022) also attempted to use leaf nutrients in order to explain the broad geographic trends in autumn leaf colour geography as emphasized by Renner and Zohner (2019), demonstrating that modern eastern North American soils exhibit significantly lower N and P content compared with Europe, with east Asia exhibiting intermediate values (table 1, figure 2 in Hughes et al., 2022). However, it is clear from their figure that in all three continents, higher soil nitrogen levels are much more common in colder regions. This is again a clear indication that many (but certainly not all!) analyses about the distribution of autumn leaf colouration should not be done for whole continents, but rather also at habitat levels or no more than at regional ones. Hughes et al. (2022) concluded by discussing the question of the proximate *versus* ultimate causation for the evolution of autumn leaf colours, that is that low leaf nitrogen levels and high soluble sugars could serve as proximate cues for leaf reddening during autumn, along with high light and cold temperatures, although the ultimate function of autumn leaf reddening could be both defensive (anti‐herbivory) signalling and photoprotection. It is worth noting that the connection between soil nutrients and anthocyanin in autumn leaves has been recently corroborated by several studies, including Wang et al. (2022), who demonstrated that autumn‐red deciduous species exhibit significantly lower N and P in senescing leaves than autumn‐yellow species. Fataftah et al. (2021) showed that supplying nitrate to trees *via* continuous feeding directly into the trunk reduced autumn reddening in *Populus*. Renner and Zohner (2022) demonstrated that autumn anthocyanins are absent in 100% of the 81 surveyed species with nitrogen‐fixing symbionts (whereas 42% of non‐nitrogen fixers do display autumn anthocyanin).

The response by Hughes et al. (2022) also finely tuned the critical and essential aspects of light absorption and photoprotection by anthocyanins at the whole leaf level that were only partly amended by Agati et al. (2021). Hughes et al. (2022) explained why contrary to the views of Karageorgou and Manetas (2006) and Pena‐Novas and Archetti (2020a), the location of anthocyanins in vacuoles is optimal for functioning as a sunscreen: (1) because the vacuole is the largest cell component, (2) because chloroplasts migrate to vertical cell walls under strong light levels and (3) because the vacuoles effectively shade cells in lower leaf layers. Hughes et al. (2022) further explained that ROS in chloroplasts are quickly converted into H_2_O_2_ that can freely penetrate the vacuole and be treated there by anthocyanins. Renner and Zohner (2020) criticized the inclusion of evergreens in the sampling by Pena‐Novas and Archetti (2020a). Hughes et al. (2022) defended Pena‐Novas and Archetti's (2020a) inclusion of evergreens in the sampling, but suggested scoring autumn leaf colours in deciduous species based on leaf colours exhibited in their native range and not in botanical gardens outside their natural habitats (as were the cases of Renner & Zohner, 2019; and Archetti, 2009a). Archetti et al. (2009a) scored many deciduous species as green although they exhibit colourful autumn leaves in their native habitats. Hughes et al. (2022) suggested that since Archetti examined many species in the Westonbirt Arboretum in Gloucestershire, UK, the mild climate there (due to the warm Gulf Stream), or because of abundant fertilization, which is known to delay leaf senescence, his observations may reflect differences related to growing in non‐native habitats in an unknown number of species. The problem of studying species outside their natural habitats that was illuminated by Hughes et al. (2022) is true for several other studies such as Panchen et al. (2014), Zohner and Renner (2017) and Zohner et al. (2017). Hughes et al. (2022) also showed that many evergreen leaves also senesce bright yellow and red (see also tables in Lev‐Yadun et al., 2012). Hence, they suggested that, in addition to scoring such species as evergreen, they could also be scored based on leaf colour during senescence, whatever season it occurs. Hughes et al. (2022) pointed to evidence that orange autumn leaves are actually red because they produce anthocyanins, saying that Renner and Zohner (2019) misclassified them. I think that such misclassification means that all the data in Renner and Zohner (2019, 2020) are wrong, and therefore, all their conclusions that were based on wrong data are invalid. Hughes et al. (2022) proposed another reason why evergreens should be included in the discussion of autumn leaf colour, because plants with evergreen, red‐deciduous and yellow‐deciduous leaves could represent strategies along a continuous resource spectrum. Hughes et al. (2022) commented that low soil fertility has long been known to favour the evolution of evergreen over deciduous leaves, and anthocyanin production in leaves is known to correlate with a number of variables associated with evergreen leaves, for example low leaf nitrogen, low maximum photosynthesis and high leaf mass per unit area. However, many temperate and boreal evergreens are conifers (Breckle, 2002; Farjon, 2017), and their dominance over large winter frozen areas is especially related to the ability of their xylem to avoid and repair spring embolism when winter ice thawing occurs (e.g. Mayr et al., 2019; Tyree & Zimmermann, 2002). Discussing in depth the evolution and ecology of being evergreen is beyond the scope of this review, but soil fertility issues cannot explain evergreen existence or dominance in many ecologies, and being deciduous in hot tropical and subtropical regions is commonly related to either winter or summer drought (e.g. Richards, 1996; Zohary, 1962, 1973), and not only to nutrition.

Joly (2022) reanalysed the results of Pena‐Novas and Archetti (2021a) about nitrogen resorption. He used a different statistical approach and found that their result of higher nitrogen resorption in red *versus* yellow leaves is wrong. Joly (2022) found that both yellow and red autumn leaves resorb more nitrogen than green leaves, an irrelevant result, because if the photosynthetic apparatus remains intact as it is in green leaves, or even only partly intact, there is less nitrogen free for recycling. Joly (2022) explained that carotenoids also protect leaves from photo‐oxidative stress, and by this, they also help in nitrogen resorption. Joly (2022) concluded that a combination of causes may be needed for explaining autumn leaf colours and that there is evidence that anthocyanins have a photoprotective effect.

The last comment by Renner and Zohner (2022) is the most correct and important contribution by these authors concerning autumn leaf colours. Renner and Zohner's (2022) paper presented highly valuable data about the relationships between nitrogen husbandry and autumn leaf colours. Renner and Zohner (2022) compiled a list of 126 deciduous tree and shrub species, belonging to 55 genera and 22 families. Eighty‐oner of these species, belonging to 31 genera in eight families, have symbiotic nitrogen‐fixing bacteria. Most of the nitrogen fixing species (49 species) belong to the Fabaceae. None of the nitrogen‐fixing species had red autumn leaves, 58% of them had yellow autumn leaves, and 42% stayed green. Of the non‐nitrogen fixers, 42% had red autumn leaves, 47% had yellow autumn leaves, and 11% stayed green. Renner and Zohner (2022) said that their results support the hypothesis that nitrogen limitations play a role in anthocyanin production in autumn leaves. Renner and Zohner (2022) suggested that the co‐evolutionary hypothesis is not valid, and in their words should be ‘retired’, and like in their previous papers ignored all other anti‐herbivory data and hypotheses. They continued by discussing the data presented by Hamilton and Brown (2001) that examined autumn leaf colour and aphid species diversity in 262 tree species from North America and the UK, and found that species with yellow autumn leaves harboured more aphid species than species with red autumn leaves. I think that this finding by Hamilton and Brown (2001) clearly showed that red autumn leaves are better defended from herbivory than yellow ones, but Renner and Zohner (2022) ignored such a possibility. Renner and Zohner (2022) also referred to Hamilton and Brown's (2001) second finding, that is that when the analysis was restricted to taxonomically specialized aphid species, a correlation between red autumn leaves and species‐specific aphid attacks emerged, and therefore, Renner and Zohner (2022) posited that both red and yellow autumn leaves attract aphids. This was already known not to be so (e.g. Chittka & Döring, 2007; Holopainen & Peltonen, 2002; Wilkinson et al., 2002). When referring to this point, it must be remembered that many specialist arthropod species commonly sequester defensive toxic chemicals from their hosts for their own defence and for mate choice (Beran & Petschenka, 2022; Eisner et al., 2005). Since what is toxic to one animal might be harmless or even beneficial to another (Gleadow & Woodrow, 2002; Janzen, 1979; Laycock, 1978), there is no surprise that more specialist aphid taxa occupy the usually better chemically defended red‐leafed trees.

The currently last exchange in this debate was the short note by Pena‐Novas and Archetti (2022). After briefly discussing some mistakes in Renner and Zohner (2022), they repeated their previous correct conclusions that the debate and the data presented by Renner and Zohner (2022) did not disprove the co‐evolutionary hypothesis. Pena‐Novas and Archetti (2022) also repeated their earlier (Pena‐Novas & Archetti, 2020a) stand that although the photoprotection hypothesis remained plausible, again it was not confirmed by Renner and Zohner's (2022) analysis of species expressing nitrogen fixation.

## 17 LEAF ABSCISSION: AN INVOLVED MECHANISM THAT ADDS TO THE COMPLEXITY

Till now, the discussions about the functions and evolution of autumn leaf colouration did not consider the process of leaf abscission as a factor that influences the efficiency of resorption of nitrogen and other resources. Leaf abscission is positively regulated by ethylene (Addicott, 1982; Jackson & Osborne, 1970), and it follows a long and regulated process of redifferentiation of the cells of what will become the abscission zone from differentiated petiole base or leaflet base tissues in order to form a band of thin‐walled soft small cells (Addicott, 1982). The abscission zone may or may not include, in addition to the separation layer, a protective layer that seals the abscission wound against biotic and abiotic dangers. When both a separation layer and a protective layer develop, the separation layer is always distal to the protective one (Addicott, 1982). The separation layer, with its thin‐walled cells, gradually becomes a mechanically weak zone that will eventually snap. However, the snapping may occur before resorption of nitrogen and other substances has ended, for instance, as I have witnessed in Finland more than once, because of strong autumnal winds. In such cases, the litter is characterized by a certain proportion of leaves that are still partly or fully green. Therefore, when measuring resorption, the environmental circumstances of autumnal leaf abscission may influence the process and should also be considered.

## 18 ANTHOCYANINS IN RED AUTUMN LEAVES MAY DEFEND FROM COMPETITION WITH FUNGI OVER NUTRIENTS

I suggest that in addition to the above‐mentioned anti‐herbivory and physiological hypotheses, red autumn leaves may be better protected than yellow ones from fungal attacks when their fungal endophytes and fungal pathogens compete with them over the resources released in order to be resorbed by the trees before leaf fall. My suggestion originates in tropical ecology. Coley and Aide (1989) realized that leaf‐cutting ants that grow fungal colonies in their nests as a food source refrain from feeding the fungal colonies with leaves that have red undersides, and suggested that the anthocyanin‐based red underside leaf colouration defends from fungal attacks. Experimental data about defence from fungal attacks in red or black fruits (Schaefer, 2011; Schaefer et al., 2008; Schaefer & Ruxton, 2011) support this hypothesis. Further support for the anti‐fungal properties of anthocyanins was also presented by Tellez et al. (2016), who found that young red leaves in tropical Panama suffer less damage from fungal attacks than green ones, and by González‐Teuber et al. (2020), who found that endophyte fungal diversity decreased in temperate South American rainforest trees that have red leaves.

## 19 ANTI‐HERBIVORY AUTUMN LEAF COLOURATION: PART OF THE PROOF MAY BE IN THE REPELLED APHID SPECIES AND NOT ONLY IN THE APHIDS THAT ATTACK THE LEAVES

I suggest that the tests of the visual defence *via* the co‐evolutionary hypothesis as reviewed and revised in Archetti et al. (2009a) and in later studies can benefit from two additional considerations: (1) the repelling olfactory aspect of autumn leaves (e.g. Blande et al., 2010; Holopainen, 2008; Holopainen et al., 2010), which was just published at the time and therefore was not yet digested and considered. (2) I suggest adding a basic assumption when studying the anti‐herbivory roles of yellow/red autumn leaves. If yellow and red autumn leaf colours deter herbivores *sensu* Archetti (2000), Hamilton and Brown (2001), Lev‐Yadun et al. (2004), Lev‐Yadun and Gould (2007) and Archetti et al. (2009a), what should have been studied was not only the aphid species and other invertebrate taxa that attack the trees (e.g. Brown, 2005; Hagen et al., 2003; Hamilton & Brown, 2001; Sinkkonen, 2006b; Sinkkonen et al., 2012), but also the autumnal aphid and other relevant herbivore taxa occurring in the same geography that do not attack the trees. I suggest that since the insect species that attack yellow or red autumn leaves are obviously fully or partly resistant to the various leaves' defences, herbivore species that are not monophagous and are occupying the habitats of tree species with colourful autumn leaves but do not attack the trees should be considered, because they may be the ones that are repelled but were always overlooked. Many previous studies and hot debates missed that critical point and should be re‐evaluated. Not understanding that the species that attack the trees are not the only ones that should be studied caused many of the studies of the co‐evolutionary hypothesis to miss a potentially critical point. I expect that a new wave of studies of defensive yellow and red autumn leaf colouration that takes the repelled insects into consideration will result in a more realistic understanding of this enigmatic and fascinating phenomenon. This approach (studying repelled potential attackers) is probably relevant to many other defence–attack biological systems.

## 20 CONCLUSIONS

Although our understanding of the physiology, ecology, defence and evolution of the phenomenon of yellow and red autumn leaves is dramatically better now compared with our understanding in the year 2000, much more descriptive, theoretical, field and experimental work should be done in order to bring that understanding to a higher level. Doing it quickly seems to be important because if the current trend of global warming continues, the ecologies in the temperate and boreal regions may change in a way that will change various ecological balances till a new equilibrium is reached, and this will decrease our ability to study it, and influence the conclusions and understanding as well.

We certainly need a much better documentation of the species and their leaf colours, their geographic distribution, the phenology of the changes at their native habitats, the anatomy of leaf reddening and yellowing, the genetic and epigenetic factors involved, the chemistry of the changes, the volatiles, the involved insects and fungi. Similarly, concerning the herbivores, we should know about the predation or parasitism of the relevant herbivores. Taxa such as *Populus*, for which we have good genetic and genomic information and which can express yellow autumn leaves, red autumn leaves or even a mixture of leaf colours within the same individual (Figures 3 and 4), can serve as a good model tree for experimentation. However, because taxa differ in their defensive chemistry and physiology, and in their herbivores and pathogens, additional taxa with such good genetic knowledge (something expected to be realistic in the near future) are required to serve as model plants.

Because yellow and red autumn leaf colouration simultaneously serve several physiological and defensive functions, and because the relative importance of these functions certainly changes with time within one tree, one leaf, each cell, within one autumn, in different autumns and under different ecological conditions, considerable effort is needed in order to better understand that dynamic mosaic. It is clear that there will not be a single and simple unifying explanation for the evolution of all yellow and red autumn leaves. Trying to look for such a simple explanation seems to be a waste of time and resources.

Studying all the above at the ecosystem, taxon, genotype, organism, leaf, cellular and molecular levels is an enormous research effort. Doing it will take many decades, so the sooner, the better.

## CONFLICT OF INTEREST

There is no conflict of interests.

### PEER REVIEW

The peer review history for this article is available at https://publons.com/publon/10.1111/jeb.14069.

## Data Availability

Data sharing not applicable ‐ no new data generated.
